# Using Sequence Similarity Networks for Visualization of Relationships Across Diverse Protein Superfamilies

**DOI:** 10.1371/journal.pone.0004345

**Published:** 2009-02-03

**Authors:** Holly J. Atkinson, John H. Morris, Thomas E. Ferrin, Patricia C. Babbitt

**Affiliations:** 1 Graduate Program in Biological and Medical Informatics, University of California San Francisco, San Francisco, California, United States of America; 2 Institute for Quantitative Biosciences, University of California San Francisco, San Francisco, California, United States of America; 3 Department of Pharmaceutical Chemistry, University of California San Francisco, San Francisco, California, United States of America; 4 Department of Biopharmaceutical Sciences, University of California San Francisco, San Francisco, California, United States of America; Georgia Institute of Technology, United States of America

## Abstract

The dramatic increase in heterogeneous types of biological data—in particular, the abundance of new protein sequences—requires fast and user-friendly methods for organizing this information in a way that enables functional inference. The most widely used strategy to link sequence or structure to function, homology-based function prediction, relies on the fundamental assumption that sequence or structural similarity implies functional similarity. New tools that extend this approach are still urgently needed to associate sequence data with biological information in ways that accommodate the real complexity of the problem, while being accessible to experimental as well as computational biologists. To address this, we have examined the application of sequence similarity networks for visualizing functional trends across protein superfamilies from the context of sequence similarity. Using three large groups of homologous proteins of varying types of structural and functional diversity—GPCRs and kinases from humans, and the crotonase superfamily of enzymes—we show that overlaying networks with orthogonal information is a powerful approach for observing functional themes and revealing outliers. In comparison to other primary methods, networks provide both a good representation of group-wise sequence similarity relationships and a strong visual and quantitative correlation with phylogenetic trees, while enabling analysis and visualization of much larger sets of sequences than trees or multiple sequence alignments can easily accommodate. We also define important limitations and caveats in the application of these networks. As a broadly accessible and effective tool for the exploration of protein superfamilies, sequence similarity networks show great potential for generating testable hypotheses about protein structure-function relationships.

## Introduction

**Figure 1:**
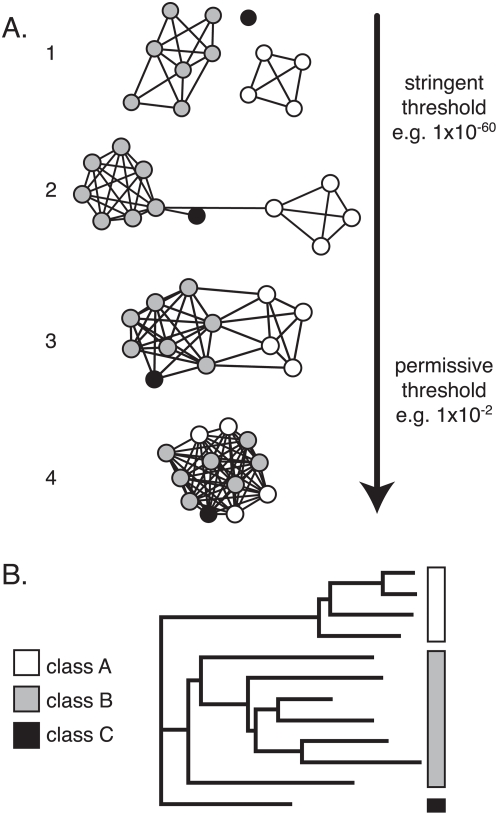
Sequence similarity network topology changes in a predictable way with the stringency of the threshold. A. Thresholded sequence similarity networks represent sequences as nodes (circles) and all pairwise sequence relationships (alignments) better than a threshold as edges (lines). The same network, depicting three simulated protein classes, is shown here at four different thresholds. At stringent thresholds, the sequences break up into disconnected groups; within each group the sequences are highly similar. The relative positioning of disconnected groups has no meaning, while the lengths of connecting edges tend to correlate with the relative dissimilarities of each pair of sequences. As the threshold is relaxed and edges associated with less significant relationships are added to the network, groups merge together and eventually become completely interconnected. B. Simulated dendrogram for a sequence set that might give rise to the network in A.

Over the past two decades, there has been a disorderly explosion of biological data, exponentially increasing in volume with time. To keep pace with the broad classes of new sequence, structural, and functional data arising from compilations of genomic and proteomic data in particular, many powerful approaches have been developed for unearthing meaningful themes and hypotheses from within the jumble. Yet there is still a critical need for improved techniques enabling fast and comprehensive analysis of large sequence data sets, especially to access the biologically useful context that can be extracted from this information. There is a particular demand for easy-to-use techniques to aid experimental biologists in finding useful starting points for analyzing diverse superfamilies of proteins. Here we address one of these techniques, sequence similarity networks (Fig. 1). A relatively new application of methods commonly used to summarize protein-protein interactions on a large scale[1], sequence similarity networks—here, in which the interrelationships between proteins are described as a collection of independent pairwise alignments between sequences—represent an attractive adjunct approach to multiple sequence alignments and phylogenetic trees. Moreover, they offer several important capabilities unavailable to these methods. First, they provide a fast and easy to compute framework for observing relationships among very large sets of evolutionarily related proteins; more importantly, when visualized they also allow the perception of trends in orthogonal information—viz., function-related information—mapped onto the context of sequence similarity. Because they provide access to these relationships in an intuitively accessible manner and are easy to create and manipulate, these networks fill a need that is not currently well-addressed by other tools. By enabling the visualization of extremely large sets of related sequences, networks provide advantages unmet by phylogenetic trees, particularly in showing all relationships that score above a user-defined similarity cut-off rather than only the small number of optimally scoring connections. Also, for the same amount of computation, a much larger set of sequences can be analyzed using a network than could be used to infer a tree. Furthermore, there are restrictions on the number of sequences that can be usefully considered in generating a multiple sequence alignment, in part due to the practical limitations of viewing alignments of hundreds of sequences. The corresponding benefit of visualizing a sequence similarity network, rather than analyzing it numerically, is that the displayed network can be overlaid with as many types of derived and orthogonal information as spring to mind. The network can then be interactively explored to see how these different features coalesce into trends (or don't) when viewed in the context of sequence similarity. Additionally, using interactive software to visualize the networks (e.g. [1]) and to link to other types of information such as three-dimensional structures (e.g. [2]) allows the evaluation of individual and sets of edges, enabling an informed researcher to decide how much to trust the relationships implied by the network structure.

There has already been a great deal of interest in generating sequence similarity networks. Enright and colleagues recognized that visualizing a network of protein similarity information[3] was a useful extension to basic protein sequence clustering methods (e.g. BLASTCLUST[4] and cd-hit[5]). They then used the MCL algorithm—designed for clustering very large networks—to identify natural sequence similarity “families” (ideally, rough functional classes) in a network of the protein universe[6]. A number of other groups followed with innovative approaches to cluster all known proteins and visualize them as attractive, enigmatic maps (e.g. [7]). More recently, there have been efforts to use sequence similarity networks for more discrete sets of related proteins[8], and PFAM has released its classification of families into the more general *clans*, creating many three-level hierarchies bundling sequences into families, and families into clans[9]. Work by Medini et al.[10] began with a sequence similarity network of the protein universe, but also isolated one small and interesting region of the network. Using more careful analyses, they made inferences about the evolution of specific protein families from the isolated region. In our own work, we have begun to use sequence similarity networks to provide context for the analysis of individual proteins that are members of superfamilies[11], to show the relative outlier status of specific functional classes within a large superfamily[12], [13], and to illustrate the correlation with lineage of conservation patterns for active site residues in a specific family of enzymes[14].

But before sequence similarity networks can be adopted for broad use, it is important to understand their strengths and weaknesses. In particular, these types of networks need to be validated in comparison to better-understood approaches. A primary motivation of this work is to address whether there is a compelling quantitative argument that sequence similarity networks can competently depict sequence similarity relationships, allowing them to be used as a framework to guide hypotheses about functional relationships. Although it has long been recognized that sequence similarity is an imperfect proxy for functional similarity, a fundamental dogma of structural biology—that sequence conservation infers structural conservation, which in turn implies functional conservation—has been extensively and effectively applied to infer functional properties on every scale. Consistent with this view, our results demonstrate that visualized sequence similarity networks perform well in representing sequence similarity information, and indeed the visualized relationships correlate well with known functional relationships. In contrast to the formal network representations of sequence similarity represented by previous studies describing algorithms for network generation, we have shown how well the displayed relationships reflect various measures of sequence and evolutionary distance, using relevant examples and quantitative assessments. Additionally, we introduce a concept: the most valuable feature of sequence similarity networks is not the optimal or most accurate display of sequence similarity, but rather the flexible visualization of many alternate protein attributes for all or nearly all sequences in a superfamily. To illustrate the results, we have used three well-studied superfamilies with nuanced functional annotations. This work is especially applicable to the study of individual superfamilies, and is complementary to previous work in this area that typically shows that networks can group all known proteins in agreement with broad definitions of functional similarity (e.g. [15]).

Here we demonstrate, using example data sets of G-protein coupled receptors (GPCRs), kinases, and the crotonase superfamily of enzymes, that sequence similarity networks recapitulate much of the information present in phylogenetic trees, that the relationships implied by networks are in agreement with known sequence and structural relationships, that networks incorporate a number of practical benefits that improve on current techniques for relating sequences, and finally, that visualization of similarity networks enables the perception of trends from the context of sequence similarity, initiating fruitful hypotheses. Finally, we report a new result relevant to the evolution of domain variation in the crotonase superfamily of enzymes that was obtained from analysis of sequence similarity networks.

## Results and Discussion

Our results provide validation of sequence similarity networks for establishing family or superfamily context and for illustrating important applications. The first two sections provide quantitative evidence to support our claim that two-dimensional distances in visualized networks correlate well with the underlying distances in high-dimensional space and with distances depicted by phylogenetic trees, indicating that the depictions are mathematically reasonable and comparable to an accepted standard. The next sections address the practical benefits we have found for sequence similarity networks in capturing known (and novel) sequence and structural relationships, and in providing different and new information compared to conventional methods for relating sequences. We also describe some of the important advantages this view of sequence similarity context provides for hypothesis generation about structure-function relationships. This latter application is most powerful when nodes in the network are painted with structural or functional information that is orthogonal to homology-based information. An example is provided by mapping sequence length and taxonomic information onto the crotonase superfamily network, leading to the discovery that there are three major groups within the superfamily that are differentiated by domain organization and that track with primary branching across the tree of life. Each section is accompanied by a brief discussion of the controls and caveats we have found to be important for effective use of this method.

### I. Visualized sequence similarity networks are satisfactory depictions of high-dimensional similarity relationships

Graph layout algorithms project the N-1 dimensional data structure into two (or three) dimensions for visualization, with the aim being to preserve, as well as possible, the actual pairwise distances between nodes in high dimensional space. In this case, the graphs are made up of nodes (sequences) connected by edges (pairwise similarity relationships). The layout used in this work, the Organic layout[16], [17] available in Cytoscape 2.6[1], uses only node connectivity to illustrate groups and inter-group relationships. This makes it suitable for visualizing thresholded sequence similarity networks, where the high-dimensional graph is defined by all pairwise sequence alignments that are better than a chosen cut-off. Because mutual sequence similarity within a protein family and the number of similarity relationships better than a threshold appear to be highly correlated, the Organic layout is able to calculate relative distances in two dimensions that are remarkably close to the underlying, mathematically ideal distances in high dimensional space, without explicitly incorporating numeric edge weights into the algorithm. (See Fig. 1 and Table S1.) An alternative layout algorithm incorporating edge weights performed slightly worse (Fig. S1). Across all of the test sets used in this work, the correlation between displayed distances and the mathematically ideal distances defined by BLAST E-values ranges from a low of 0.838±0.002 to a high of 0.936±0.003; the variation in correlation appears to be associated with variations among the specific sets of proteins analyzed, data set curation, and the selected E-value cut-off rather than with the size of the network in terms of nodes and edges (data not shown). The visually discernable clusters tend to overlap with sequence clusters as determined by related approaches, such as the NCBI BLASTCLUST program (See Fig. S2).

Additionally, we found high correlations between a Class A GPCR network composed of 605 sequences and networks from this set where 20% of the sequences were removed at random. To address the impact of missing data on network topology, we compared the laid-out distances between sequences present in the full network and these 80% networks (Fig. S3). Here, the average correlation is 0.892 with a standard deviation of 0.016, indicating that the observable distances are very similar. The underlying BLAST-defined distances also remained extremely similar, at 0.993±0.004. The observable distances for the 80% network were also very close to the 80% BLAST-defined distances (0.901±0.010) as well as to the underlying BLAST distances for the full sequence set (0.894±0.014). Thus, the implied sequence interrelationships do not depend strongly upon the presence of specific sequences.

### II. Sequence similarity networks recapitulate much of the information present in phylogenetic trees

**Figure 2:**
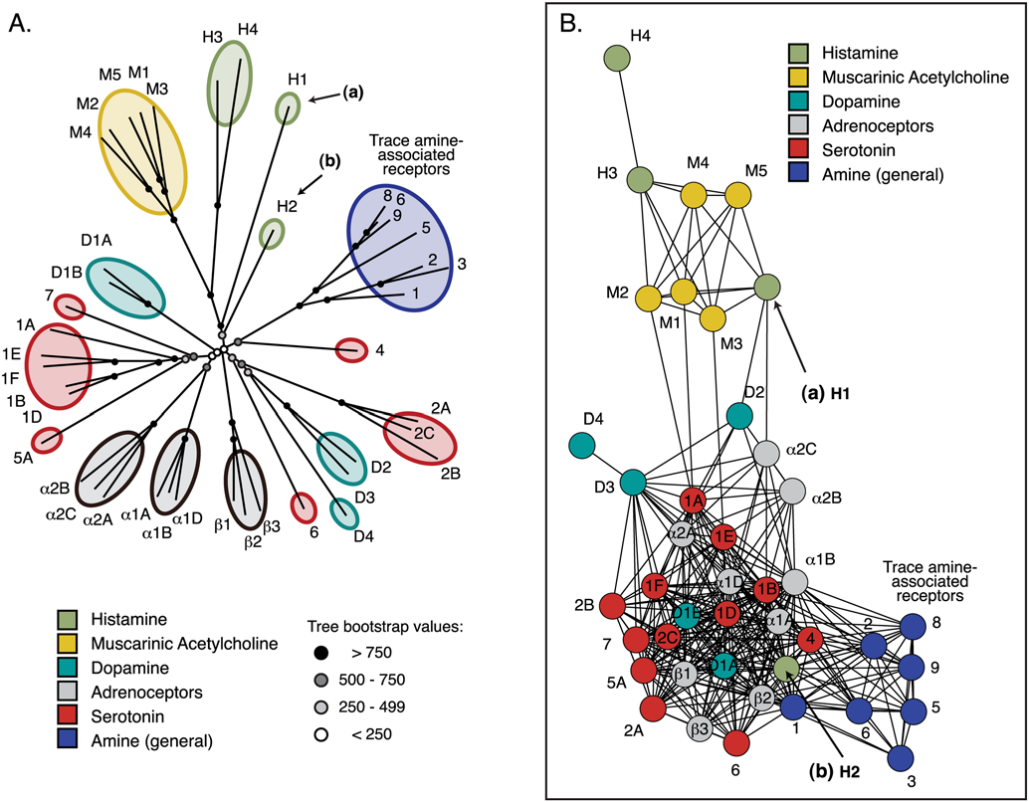
Comparison of trees and networks: amine-binding GPCRs. A. Neighbor-Joining tree describing the interrelationships of 42 amine-binding human GPCR domains. Sequences are labeled according to the common name for their class (e.g., the sequence labeled α1D is adrenoceptor α1D; see additional data file 5 for all sequence database identifiers). B. Sequence similarity network including the same 42 sequences as in (A). This network was thresholded at a BLAST E-value of 1×10^−33^: only edges associated with E-values more significant than 1×10^−33^ are included in the network. This network contains 324 edges; the worst edges displayed correspond to a median of 30% identity over an alignment length of 280 amino acids. See Table I for a quantitative comparison of the two representations. The sequences labeled (a) and (b) are discussed in the text.

We examined the similarity relationships implied by phylogenetic trees and networks of two small protein families (amine-binding GPCRs, and the STE and WNK kinases) and the kinase superfamily. Both sequence families are simple to align—highly conserved transmembrane helix domains anchor the amine-binding GPCRs, while the STE and WNK kinases have an average percent identity of 36% across the alignment. The distances between sequences in a neighbor-joining tree of the 42 human amine-binding GPCRs and the corresponding sequence similarity networks are well correlated (R = 0.712; see Table 1); notably, with this set of proteins annotated by their ligands, the network does as good a job of grouping functionally-similar sequences as the tree. As can be seen in the neighbor-joining tree in Fig. 2A, most of the clades are about equidistant from one another, with the exception of the muscarinic acetylcholine group, which is slightly more similar to a pair of histamine-binding GPCRs (H3 and H4). These comparatively longer branches are demonstrated in the network, and the intermediate characteristics of a third histamine-binding GPCR, labeled (a) in Fig. 2, are captured both in the tree and the network. A fourth histamine-binding GPCR, labeled (b) in Fig. 2, is closer to the central branch point of the other amine GPCRs than any other sequence in the tree. Accordingly, it is embedded in the larger amine group that is closer to the central branch point relative to the muscarinic acetylcholine and three histamine class sequences in the network. A similar level of correlation was found between trees and displayed distances (0.714) in 51 human STE and WNK kinases, and qualitative features were mirrored as well. Both the tree and the network clearly demonstrate the outlier status of the STE20: STLK kinase domains (labeled (a) in Fig. S4). See Table S2 for statistics on these kinases.

**Table 1:** Comparison of mathematically ideal and displayed pairwise network distances between 42 human amine-binding GPCRs.

A. BLAST E-values (from pairwise alignments)	A. BLAST E-values		
B. Organic layout	R: 0.906±0.034		
	Z: 11.87		
	P: 8.04×10^−33^	B. Organic layout	
C. Neighbor Joining tree	R: 0.758±0.034	R: 0.712±0.034	
	Z: 9.91	Z: 9.43	
	P: 1.95×10^−23^	P: 2.14×10^−21^	C. NJ tree
D. Distances from multiple sequence alignment	R: 0.715±0.034	R: 0.645±0.034	R: 0.944±0.034
	Z: 9.11	Z: 8.24	Z: 13.07
	P: 4.14×10^−20^	P: 8.47×10^−17^	P: 2.29×10^−39^

Pearson's correlations (R) and associated Z-scores (Z) and P-values (P) describing the similarity between the relative pairwise distances between 42 amine-binding GPCR domain sequences as assessed by (A) all shortest paths between −log_10_(BLAST E-values), (B) the shortest paths between sequences as displayed by a two-dimensional graph layout algorithm, (C) the shortest paths between sequences in a Neighbor-Joining tree, and (D) the relative pairwise distances calculated from a multiple sequence alignment. Additionally, pairwise BLAST E-values and the graph layout algorithm correspond to a network thresholded at an E-value of 1×10^−33^. Note that the network layout (B) is a visual representation of the underlying distances in (A), while the tree (C) is a visual representation of the underlying distances in (D). A and D cannot be visualized exactly in fewer than N-1 dimensions.

**Figure 3:**
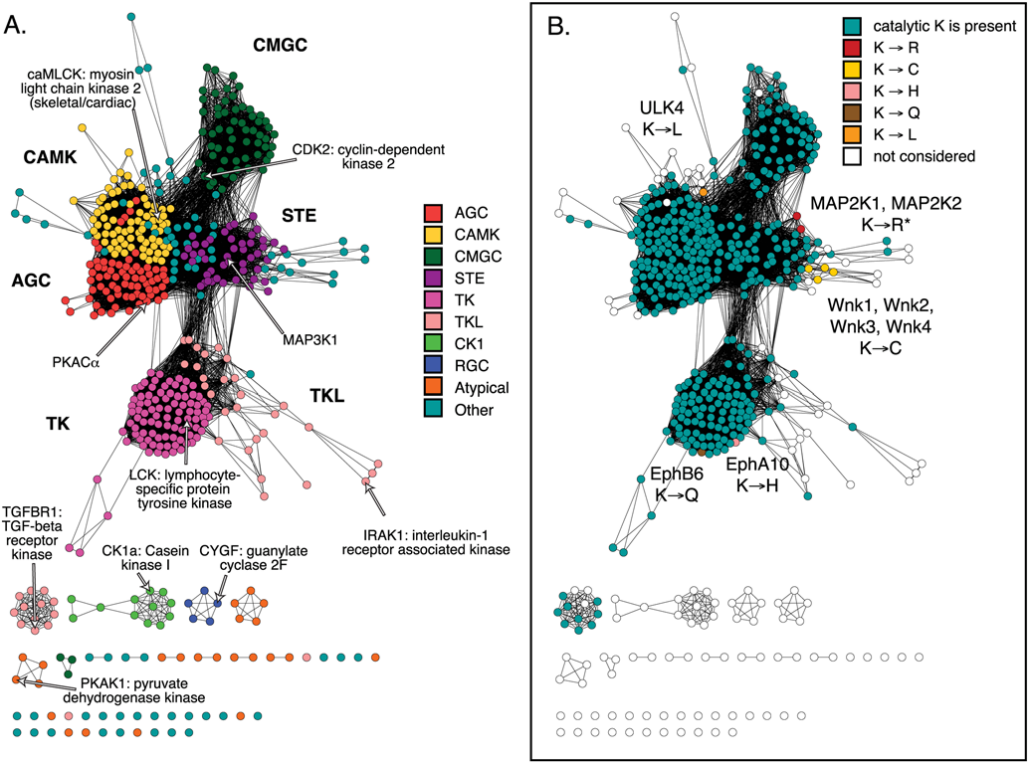
Sequence similarity networks are useful tools for exploration of the kinase superfamily. Two ways of coloring the same network of 513 human kinase domains are shown. The network is thresholded at a BLAST E-value of 1×10^−25^. The worst edges displayed correspond to a median of 29% identity over alignments of 260 residues. A. Network colored by kinase class. B. Network colored by the presence of a catalytic Lys in the “VAIK” motif: Each of the 513 sequences was aligned to a sequence model of the kinase domain, and the identity of the residue at the catalytic Lys position is mapped to the network. *Note that MAP2K1 and MAP2K2 registered a Lys to Arg substitution due to a sequence alignment error. The other labeled kinases truly do not contain a homologous catalytic K, but only the WNK kinases have been shown to have kinase activity. See Table II for statistics.

In order to assess the correspondence between a very large phylogenetic tree and sequence similarity networks, we used a dendrogram of the human kinome[18], which uses sequence similarity to classify all of the kinase domains in the human genome into a number of broad classes. This tree depicting the classification of each kinase has been enormously useful to researchers since being published; in particular, it gives a sense of how a kinase of interest relates to all others. Although the pairwise relationships between the CK1 kinase class and the other canonical kinase domains are not significant enough to be connected at the E-value threshold chosen for Fig. 3, the pairwise distances between the large connected group are still strongly correlated with the distances in the seminal Manning kinase tree[18] (R is 0.628 when comparing the laid out distances in the connected cluster in Fig. 3 to the tree distances for the 419 sequences in common from the full Manning tree, which contains 491 kinase domains; see Table 2 for more statistics).

**Table 2:** Comparison of network and phylogenetic tree distances between 419 kinase domains.

A. BLAST E-values	A. BLAST E-values	
B. Organic layout	R: 0.934±0.003	
	Z: 41.2	
	P: 0.0	B. Organic layout
C. Manning et al. 2002 human kinome tree	R: 0.683±0.003	R: 0.628±0.003
	Z: 39.5	Z: 40.0
	P: 0.0	P: 0.0

Pearson's correlations (R) and associated Z-scores (Z) and P-values (P) describing the similarity between the relative pairwise distances between 419 human kinase domain sequences in common as assessed by (A) all shortest paths between −log_10_(BLAST E-values), (B) the shortest paths between sequences as displayed by a two-dimensional graph layout algorithm, and (C) the shortest paths between sequences in the phylogenetic tree published in Manning et al. 2002[18]. The pairwise BLAST E-values and the graph layout algorithm correspond to a network thresholded at an E-value of 1×10^−25^.

**Figure 4:**
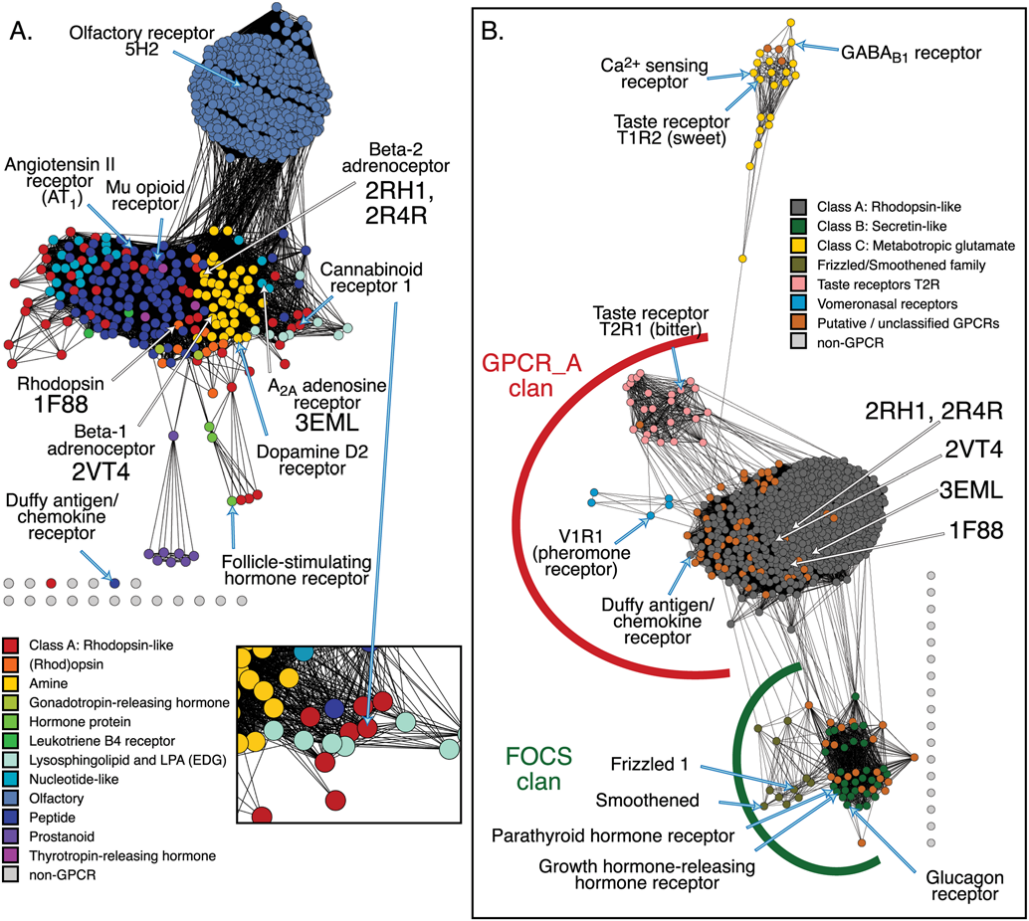
Visualizing very distant interrelationships between GPCRs. A. 605 human Class A: Rhodopsin-like GPCR domains. This sequence set includes the 42 amine-binding sequences from Table II and Fig. 2. This network was thresholded at a BLAST E-value of 1×10^−11^; the worst edges displayed correspond to a median of 24% identity over an alignment length of 210 amino acids. Sequences colored red for “Class A: Rhodopsin-like” were not classified to a specific subgroup within the class. B. 766 human GPCR domains. This sequence set includes all of the 605 Class A sequences from (A), now colored dark grey. This network was thresholded at an E-value of 1×10^−2^, and the worst edges displayed correspond to a median of 22% identity over an alignment length of 120 amino acids. Also included in both networks is a set of 17 sequences in light grey. These sequences were used here as negative controls, and were randomly drawn from the human proteome and not annotated as GPCRs. The red and green clan labels correspond to PFAM clans[9]. The sequences that are associated with or that are extremely similar to high resolution structures are noted [PDB identifiers 1F88[22], 2VT4[23], 3EML[26], 2RH1[24], and 2R4R[25]]. See Table S1 for network statistics.

Note that while there are many similarities between the interpretations that can be made from the information provided in a network and a tree, phylogenetic trees are based on an explicit evolutionary model that is missing from sequence similarity networks. Thus, networks are not an adequate alternative to a tree, as the interrelationships they depict cannot be used as a basis for inferring evolutionary history. Indeed, there is a fundamental difference between the network composed of nodes representing contemporary protein sequences that may be connected with cycles, and the acyclic Steiner tree with introduced ancestral nodes that can be used to describe a phylogenetic tree. Despite this, and particularly in the case of large networks with many edges, we have found anecdotally that the composition of many independent alignments as a graph projected into two dimensions enables a visual estimate of confidence in a displayed group-wise similarity relationship—a single edge representing a pairwise alignment at 22% identity may look like noise, but a large number of edges representing slightly different 22% identity alignments between different members of the same two discrete groups can be more convincing, particularly when there are known structural and functional relationships between the groups, as in the GPCR networks depicted in Fig. 4. Thus, by including many more relationships than are possible in a tree, we speculate that networks can assist in separating sequence similarity signal from noise.

### III. The relationships implied by sequence similarity network topology agree with known sequence and structural relationships

The structural relationships between different functional classes of GPCRs can be extremely distant. At the low stringency threshold at which inter-group relationships can be visualized using networks, many of the displayed edges represent poor alignments. In Fig. 4A, all of the human “Class A: Rhodopsin-like” GPCRs are shown at an E-value cut-off chosen to demonstrate the relationships between the major subgroups of this class. The largest known mammalian gene family[19], the olfactory receptors (OR), clearly forms a distinct group of its own. There are 252 edges linking the ORs to the other Class A sequences, representing inter-group pairwise alignments ranging from E-values of 1×10^−16^ (24% identity across 305 residues) to 9×10^−12^ (31% identity over 121 residues). None of these alignments can be expected to be error-free, but the fact that there are so many between the same two groups, and that sequence and functional relationships have been established for decades[20] implies that the existence of the edges—if not the details of the alignment underlying each individual edge—is reliable. The absence of edges between the Class A GPCRs and a number of decoy “non-GPCRs” is a further check to help evaluate whether or not to trust the implied similarity relationship. Note that this data set is too large to use in generating a phylogenetic tree using conventional methods.

One important application of sequence similarity networks is using them to form general functional hypotheses for sequences whose molecular functions are unknown. A typical protein superfamily sequence set contains a number of well-known families or characterized groups, as well as other groups that can be confidently classified to the superfamily but which are uncharacterized or for which the evidence for annotation with a more specific family label does not exist. In Fig. 4A, those sequences are represented as the red “Class A: Rhodopsin-like” sequences; in 4B, they are represented as the orange “Putative/unclassified GPCRs.” Clearly, the visualized network gives more information about how these sequences fit into the larger context of the superfamily than can be conveyed by a listing of scores or even a multiple alignment or tree. One relatively well-characterized Class A GPCR, Cannabinoid receptor 1 (CB1), is not associated with a more specific ligand class label in this data set, but is nestled in among a number of “Lysosphingolipid and LPA” sequences; this group is unsurprisingly involved in lipid signaling. As the literature shows that endogenous ligands for CB1 are also involved in lipid signaling[21], if CB1 had been uncharacterized, the network topology would have given hints about which sort of ligand class might lead to activation of the protein.

Another feature accessible from the network representation is so basic that it is easy to overlook—networks enable the conversion of lists of labeled protein sequences to a visually intuitive display of the entire data set. Thus, even given the caveats, the network shown in Fig. 4A provides a view of broad relationships across the rhodopsin-like GPCRs that is informative in a way not accessible from multiple alignments or trees. Of added interest, this view shows how the well-characterized rhodopsin sequence—one of the scant handful of GPCRs associated with a high resolution structure[22], along with the beta-1 and beta-2 adrenoceptors[23]–[25] and A_2A_ adenosine receptor[26]—fits into the context of the entire class, helping to illustrate its relative usefulness for making inferences about other subgroups in this network. (The other existing GPCR structure—rhodopsin from squid[27]—is distant from the human rhodopsins and is not shown in Fig. 4.) In Fig. 4B, six additional classes from the multiple GPCR superfamilies have been added to the analysis, and in order to observe group-wise connections, the threshold has been scaled back to a statistically insignificant E-value of 1×10^−2^. While the different classes have long been known to be functionally and structurally related, as recently as 1999 the different groups were described as having no sequence similarity[28]. Even more than in 4A, the most distant alignments in 4B are expected to have errors; in particular, the single edge between the Class A and Class C sequences in 4B, representing a 22% identity alignment over 135 amino acids, potentially represents a specious connection that should be undetectable from sequence information alone. However, despite the serious limitations in using such low significance sequence similarity scores for prediction of functional properties, other evidence exists to suggest that this is a useful representation of the GPCR superfamily. Except for the Class C group, the group-wise clustering is in line with the PFAM clan grouping. PFAM clans represent higher-order groupings of PFAM family models[9]: the GPCR_A clan includes “Class A: Rhodopsin”, “Taste receptors T2R”, and “Vomeronasal receptors”; the FOCS clan includes “Class B: Secretin-like” and the “Frizzled/Smoothened family”; and the “Metabotropic glutamate” group is not included in a clan. This example suggests an important rule of thumb for the use of sequence similarity networks: connections among distant sequence groups based on statistically insignificant scores should not be used for functional prediction without expert knowledge of a system; however, when expert knowledge is available, even poor significance networks may provide useful information for understanding the distribution of a large related sequence set.

### IV. Sequence similarity networks incorporate practical benefits that improve on current techniques for relating sequences

Not only do sequence similarity networks retain the basic clustering and topology information present in phylogenetic trees, but they may also be a better representation—for the purposes of developing hypotheses about protein family sequence and structural interrelationships—than phylogenetic trees. Whereas a phylogenetic tree requires the complexity of all of the pairwise relationships in a multiple sequence alignment to be projected down into one dimension, a sequence similarity network can show multiple neighbors for a given sequence. In so doing, the network can reveal sequences that may have sequence characteristics useful for linking divergent clusters in multiple alignments.

Additionally, it is not necessarily appropriate to include a sequence in a multiple sequence alignment that is firmly in the twilight zone of sequence similarity relative to most of the other sequences in the alignment[29]. A thresholded sequence similarity network allows the researcher to define the minimal level of similarity that is acceptable for use in analysis, and transitive relationships still allow the observation of group-wise similarity without diluting the signal from other more significant relationships. The fact that similarity networks are not based on a single multiple sequence alignment is an important advantage: a good multiple sequence alignment can be very difficult to construct in the case of a large or diverse sequence set. And from a practical standpoint, while it can take weeks or months to curate a global multiple sequence alignment and then wait for phylogenetic inference software to converge on a tree of reasonable quality, all of the networks discussed in this work took between a couple of minutes and a couple of hours to generate on a laptop computer. Researchers can also take advantage of existing resources that facilitate the mapping and interactive visualization of large collections of annotations and protein descriptors used to color network displays (ref. [1]). This level of flexibility is unavailable in any commonly used tree viewing software.

### V. Visualized sequence similarity networks enable the perception of trends from the context of sequence similarity, leading to fruitful hypotheses

The context provided by the similarity network can be exploited in many ways. For example, the kinase networks shown in Figs. 3A and 3B differ only in the functional properties by which they are colored. By coloring nodes according to the identity of an important catalytic or specificity position, sequence similarity networks show a clear utility in tracking functional changes with a protein subfamily. In Figure 3B, the coloring exposes functional outliers; nodes in the kinase superfamily are colored blue-green if a lysine is present at the appropriate position for binding and orienting the alpha and beta phosphates of ATP within the kinase domain (the “VAIK” motif lysine). While the vast majority of kinase domains clearly demonstrate the expected presence of this important catalytic position, there are a number of salient exceptions—in particular the catalytically active With No Lysine (WNK) kinase, in which the catalytic lysine accomplishes its role using a different subdomain[30], [31]. The other kinases without the catalytic lysine were recently described as pseudokinases[32].

**Figure 5:**
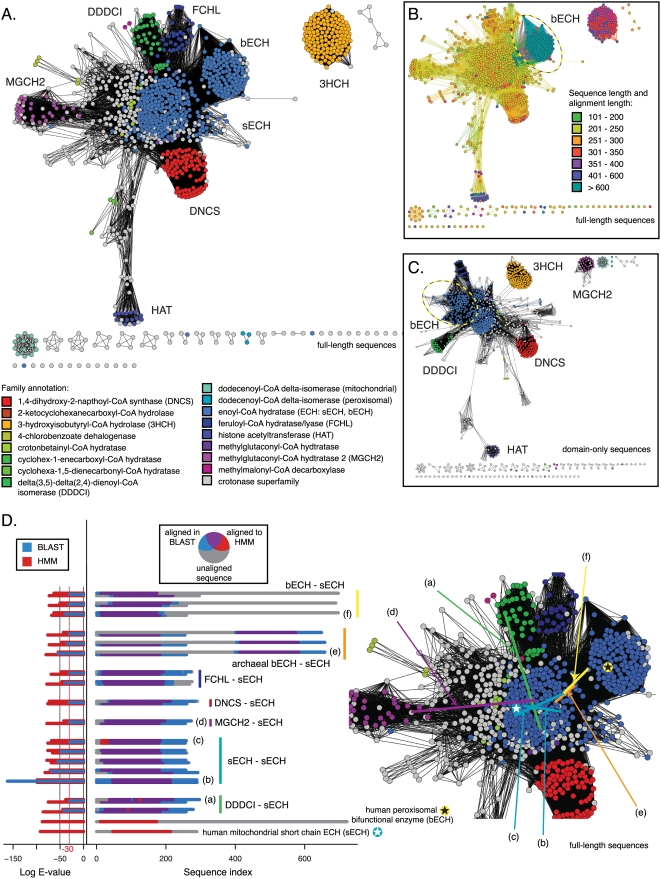
Crotonase superfamily: sequence similarity network from full-length sequences and from just the domain in common. The displayed networks all describe the pairwise relationships between 1,170 sequences from the crotonase superfamily. A. Network colored by family annotation, involving full-length sequences, thresholded at an E-value of 1×10^−30^. The worst edges displayed correspond to a median of 33% identity over alignments of 250 residues. B. The full-length network from A with nodes colored by sequence length and edges colored by alignment length. The same bifunctional enoyl-CoA hydratases (bECH) are marked with a dashed oval in B and C. C. Network colored by family annotation, involving just the crotonase domain, thresholded at 1×10^−29^. The worst edges displayed correspond to a median of 38% identity over alignments of 180 residues. D. 17 selected edges from the network in A and B. In the left panel, for each pair of sequences participating in an alignment, the log E-value versus the HMM used to define the crotonase domain is shown for each sequence—the single domain ECH (sECH) is on the bottom, and the second member of the pair is on the top—and the log BLAST E-value for the alignment between the two is in the middle. Two example bECH and sECH sequences (not alignments) are shown at the bottom of the left and middle panels. In the middle panel, each amino acid in each sequence from the 17 alignments is colored according to whether it was aligned to the crotonase domain defined by the HMM, and/or was paired to the other sequence in the BLAST alignment used to define an edge. Locations of six of these edges are marked in the enlarged view of the network in A in the right panel. The locations of the example bECH and sECH sequences are marked in the right panel using stars. See Tables S1 and S3 for quantitative comparisons.

In the course of considering the effect on network topology from using full-length sequences or only single domains, new groupings for the enoyl-CoA hydratase family were revealed, based on changes in domain architecture. (The enoyl-CoA hydratase family (ECH) is the constituent family for which the larger ECH superfamily was named.) Most proteins are composed of two or more domains, and the combination of multiple domains may modify the function of a multidomain protein relative to its single domain homologue[33]. A comparison of full-length and single domain sequences is especially relevant for highly divergent superfamilies in which domain organization may vary across different subgroups and influence network topology. Using the ECH superfamily (also known as the crotonase superfamily)[34] for these tests, we found that there can be a large degree of qualitative and quantitative correspondence between full-length sequence networks and domain-only networks when BLAST is used as a similarity metric, thanks to the fact that it calculates local alignments. Since the edges are representations of the local regions of similarity between sequences, as demonstrated in the agreement between Fig. 5A and 5C, the topology information does not change dramatically, whether the domain in common is isolated, or is embedded in a larger, multi-domain sequence (the correlation in displayed distances between the two networks is 0.868; see Table S3 for full statistics). However, in the domain-only network, the alignments leading to a similar topology are shorter and have higher sequence similarity, leading to differences in the associated E-values. Importantly, the sequence region defined as the crotonase domain by a hidden Markov model (HMM) and the region covered by the BLAST alignment are largely overlapping; see Fig. 5D for an illustration of selected alignments used to define edges from the full-length crotonase network. At significant BLAST alignment E-values—in particular, within the range included in the network in Fig. 5A, 5B, and 5D—the BLAST alignments tend to extend slightly beyond the crotonase domain. For the crotonase and GPCR superfamilies, the families of network topologies across a range of different E-value thresholds do not change substantially whether or not the domain-only sequence is used to construct a network with BLAST (data not shown).

While network topology is not strongly affected by sequence similarity outside the domain of interest in the ECH and GPCR superfamilies, this may not be the case with all superfamilies. In practice, we have found that better resolution can be achieved using networks of full-length sequences, as the greater variation in lengths of alignment and corresponding similarity scores allows a more nuanced discrimination between different groups of proteins. Yet this comes at a risk of including relationships that can be mistakenly attributed to the domain of interest. If an additional domain in common happens to be more conserved than the domain of interest, unexpected edges will link groups that the investigator would expect to find distant from one another. Investigators should weigh these issues and consider their familiarity with the superfamily before interpreting a full-length sequence network in the absence of a comparable single domain network. A useful control we use is to generate networks of each domain in a multidomain set and contrast the results with the network for the full-length proteins. Here, mapping lengths onto the network visualization clearly indicates the existence of domain differences in the ECH family (Fig. 5B); the general crotonase domain tends to be about 250 residues long, and there are a number of families whose full-length sequences are significantly longer, including a subset of ECHs (see the dashed ovals in Fig. 5B and 5C), 3-hydroxyisobutyryl-CoA hydrolases (3HCH), and histone acetyltransferases (HAT).

**Figure 6:**
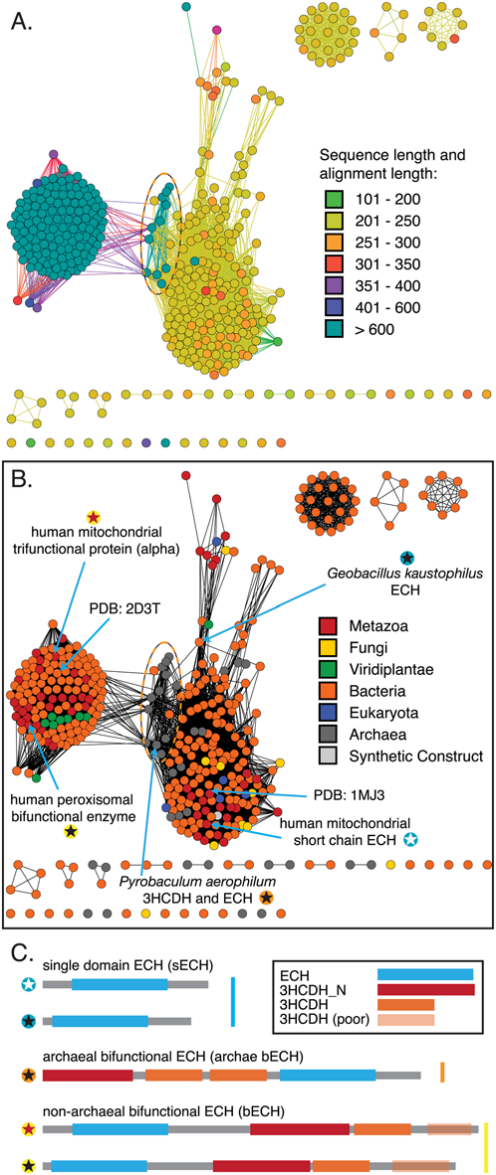
Domain shuffling in the enoyl-CoA hydratase family. The displayed networks contain all 410 enoyl-CoA hydratases from the crotonase superfamily network in Fig. 5A. The network is thresholded at a BLAST E-value of 1×10^−50^; the worst edges displayed correspond to a median of 40% identity over alignments of 260 amino acids. A. Network nodes colored by sequence length and edges colored by alignment length. B. Network nodes colored by species kingdom (Fungi, Metazoa, Viridplantae) or superkingdom (Bacteria, Eukaryota, Archaea). The same archaebacterial bifunctional enzymes are marked with a dashed oval in both A and B. C. Representative domain structures for the three major classes of enoyl-CoA hydratase-containing sequences, with domains defined using PFAM HMMs[36].

Exploration of the domain differences in the enoyl-coA hydratases—by mapping species categories onto the network—leads to new observations that have not previously been reported. We discern three major groups of ECHs: bifunctional two-domain proteins (including an ECH domain) found in bacteria, metazoans, and plants; these are variously known as multifunctional enzyme MFE-1, peroxisomal bifunctional enzyme, and the alpha subunit of mitochondrial trifunctional protein[35] (here, bECH). A second group of bifunctional proteins is found in archaebacteria (archaeal bECH), and the third group of single domain ECHs (sECH) is found in all branches of the tree of life (also known as hydratase-1). The bifunctional proteins are all more than twice the length of the general ECH domain, because they contain the N- and C-terminal domains associated with 3-hydroxyacyl-CoA dehydrogenase activity (3HCDH_N, and 3HCDH, respectively[36]); this reaction is the third step in fatty acid degradation, while ECH catalyzes the second step. The network topology in Fig. 6A and 6B indicates that the ECH domain in the archaeal bifunctional proteins is more similar to the sECH ECH domain than either of these two domains is to the ECH in the other bifunctional protein cluster; a look at the underlying alignments indicates that the domain ordering between archaeal and non-archaeal bECH sequences has been reversed (Fig. 5D, Fig. 6C). A pair of phylogenetic trees—one using full-length sequences and the other just the ECH domain from representative sequences—implies that the archaeal bECH ECH domain is most similar to the sECH domain (see Fig. S5); additionally, the significant sequence similarity between each type of ECH domain indicates that the three domain structures most likely arose through an evolutionary mechanism other than convergent evolution.

### VI. Concluding remarks

We expect that the use of sequence similarity networks may soon become as common in laboratories as the use of multiple sequence alignments. As shown here, these networks can be used to display distances that are accurate from a mathematical perspective, as well as comparing favorably to an accepted method for establishing molecular similarity, the phylogenetic tree. Sequence similarity networks reiterate known structural and functional relationships, and can be used to analyze very large data sets in a timely manner, allowing many different networks to be explored in the time required to generate a single phylogenetic tree of reasonable quality. However, we see the real promise of this technique as allowing a knowledgeable scientist to observe basic connections and clustering in a protein superfamily of interest in the context of orthogonal information. Thus, a good framework for visualizing networks performs well in recapitulating known group-wise connections and clustering. More critically, it should provide a clear view of all of the proteins in the dataset, and flexibility in mapping different features to the visual display so that large-scale and group-wise trends as well as outlier status can be discerned-the particular network layout algorithm used is not important as long as it adequately represents similarity; there are many ways a layout algorithm can be optimized to correspond more closely to some numerical ideal. Networks can be generated from protein distance data derived from many types of analyses, but for simplicity and because of the advantages of speed and the ability to use very large sets of proteins, we have used BLAST in this paper. Moreover, clustering of proteins can also be obtained in many ways. In this paper, we have used a simple method to underline the value of protein similarity networks when tagged with functional information. While we argue that coming to a final conclusion based on a pairwise BLAST alignment is generally not supportable, visualization of sequence similarity networks provides—using even such a simple metric as BLAST—an environment for exploring complex protein data sets and the straightforward generation of hypotheses to be tested using more rigorous methods. The developers of Cytoscape are actively working on extending the application to facilitate analysis of sequence similarity networks[37]; some of the features under development are automated calculation of BLAST-based similarity networks given a list of sequences, clustering algorithms for semi-automated detection of protein groups, and speed and cosmetic improvements to open-source network layout algorithms. Keeping in mind the quality of the underlying data and the caveats discussed here, we encourage the use of sequence similarity networks as a first step in analyzing diverse sequence data sets because of their potential to reveal new and unexpected relationships.

## Materials and Methods

### I. Data set sources

The human GPCR sequences and ligand-based annotations were extracted from the GPCR NaVa Database[38] on Jan. 22, 2008. This database is focused on naturally occurring variants of GPCRs; the sequences used in this work were only those associated with the 773 SwissProt identifiers corresponding to the unique gene used to group each NaVa DB set of variants.

The kinase sequences and annotations were drawn from the base set of 621 human kinase domains in Kinbase (available at http://kinase.com/kinbase)[18] on Mar. 5, 2008. The Newick-format eukaryotic kinase tree is also available at http://kinase.com/human/kinome/groups/ePK.ph)[18]. This tree was chosen for use because of its previous use in providing context for investigations of the structure-function relationship in kinases—when a researcher wishes to select some number of representative kinases, the structure of the kinome tree is often used in order to guide sampling of distinct classes, or even to show how one kinase class relates to another (e.g. [39]).

The crotonase superfamily sequences and annotations came from the 1,330 publicly available sequences in this superfamily in the Structure-Function Linkage Database[40] on Jan. 16, 2008 from http://sfld.rbvi.ucsf.edu. The crotonase superfamily represents a diverse set of homologous enzymes diverged to catalyze a range of different overall reactions using different substrates and leading to different products. Many of the member proteins have been well-characterized functionally and structurally[34], [41], making it a useful set of proteins for this analysis.

### II. Data set curation

GPCRs: To remove duplicate and highly similar sequences, the 773 GPCR sequences were winnowed to 766 by filtering to a maximum of 99% identity using cd-hit[5]. The GPCR domain—effectively the seven transmembrane helices and connecting loops—was then isolated from each sequence by extracting the region of each sequence that aligned to a hidden Markov model (HMM) of the domain. A library of three GPCR domain models was used in this step; only the best model of the three was used to define the domain. The first domain model was based on the GPCR_A PFAM clan alignment[9]; the alignment was used to train an HMM. The second domain model was based on the FOCS PFAM clan alignment, corresponding to Class B GPCRs; the alignment was clipped to isolate just the region beginning with transmembrane helix 1 and ending with transmembrane helix 7. The third model was trained on the PFAM 7tm_1 family alignment[36], corresponding to the Class C: Metabotropic glutamate group. Each PFAM alignment was downloaded on Jan. 30, 2008. All HMMs were constructed using the HMMER package hmmbuild and hmmcalibrate commands, and sequences aligned to the HMMs were extracted from the output of the hmmpfam command (package available at http://hmmer.janelia.org). An additional 20 sequences were drawn at random from the human proteome to serve as non-GPCR controls; three of these were already annotated as GPCRs and discarded. The remaining 17 non-GPCR sequences were clipped to a length of 289 residues—the median length of the GPCR domains in the 766-domain sequence set—and included in the network analysis of the two larger GPCR data sets.

Kinases: Beginning with the 621 human kinase domains, all sequences labeled as pseudogenes were removed, leaving 517 domains. The 517 domains were then filtered to a maximum of 99% identity as described above, leaving 513 sequences.

Crotonases: The initial 1,330 crotonase superfamily sequences were filtered to a maximum of 99% identity as described above, leaving 1,170 sequences. In order to define a general crotonase domain, the best-resolution structure from each applicable SFLD crotonase family[40]—1mj3, 1q52, 1nzy, 1dci, 1sg4, 1pjh, 1hzd, 1ef8—were aligned and used to generate a structure-based sequence alignment using the Chimera MatchMaker and Match->Align commands[42]. Two diverse sequences from the remote member family, the 3-hydroxyisobutyryl-CoA hydrolases, for which no experimentally determined structure is available—were themselves aligned to the structure-based alignment using the profile alignment option in MUSCLE[43]. The closely overlapping regions from the structural alignment were then used to define the borders of the crotonase domain; this region was clipped out of the combined alignment and used to construct an HMM model and isolate domain sequences from each of the 1,330 crotonase sequences as described above.

### III. Construction of networks; internal network statistics; decoys

The sequence similarity networks consist of a collection of edges corresponding to pairwise relationships that are better than a defined threshold. For this work, pairwise relationships correspond to BLAST alignments associated with an E-value[4]. The fastest way to construct the network is to use formatdb to create a custom BLAST database of a sequence set of interest, search the database with each individual sequence in the set using blastall, and treat hits to each sequence better than a threshold E-value as edges. However, by using a set of related proteins as a database, the background model assumption that similarity hits will follow an extreme value distribution is violated. Thus, while we use the BLAST E-value rather than the BLAST score to define similarity between sequences because it includes a number of helpful corrections[44], it must be considered as a type of score, rather than a true expectation value.

Additionally, BLAST E-values and scores are not symmetric—for a given comparison between two sequences, the alignment, score, and E-value can vary depending on which sequence is used as the query. In tests we performed to adjudicate this issue, we found that 74% of the comparisons in a large network have “backward” and “forward” E-values within 5 log units—regarding the other 26%, the median average log E-values begin at −46.5 and decrease as the score asymmetry increases; for our data set, alignments corresponding to log E-values of −46.5 had a median percent identity of 35% over 290 amino acids (see Fig. S6). This indicates that the greatest asymmetry is found in the better-scoring comparisons. The networks in this work use the best E-value associated with each pairwise comparison.

To aid in evaluating the networks, we create quartile plots of alignment percent identity, alignment length, and edge count versus edges binned by associated E-value (see Fig. S7). This gives a sense of how the alignments change with the E-values, and can assist in picking an informative E-value threshold. For instance, only networks based on alignments that cover at least the length of the domain in common and have greater than 30% sequence identity may be of interest. Another simple control we suggest is to add sequences known to be unrelated to the sequence data set to the network (see the discussion in Results Section III). If the selected threshold results in edges between sequences of interest and the sequences known to be unrelated, this is a clear indication that some of the edges at that threshold are at the same level of sequence similarity as background noise.

Sequence similarity networks in this work are visualized using the Organic layout[16], [17] in Cytoscape 2.6[1], with the exception of the comparison between the Organic layout and the Cytoscape force-directed layout weighted by BLAST E-value shown in Fig. S1. The Organic layout is also based on a force-directed layout algorithm; see the supplementary data website for a movie that illustrates how force-directed layouts work.

### IV. Construction of phylogenetic trees

The amine-binding GPCR tree was constructed from all 42 sequences in the “Amine” class (a subclass within the Class A GPCRs, which are themselves a subclass within the 766 human GPCR domain data set). The 51-sequence kinase tree included each sequence from the 513 human kinase domain sequence set that was annotated as an STE or WNK class kinase. Both trees were constructed using the same protocol: The sequences were aligned with MUSCLE[43]; the amine-binding GPCRs were on average 29% identical across the alignment, and the STE/WNK kinases had an average percent identity of 36%. A Neighbor-joining phylogenetic tree[45] was then inferred from the alignment using the PHYLIP 3.6 package (available at http://evolution.genetics.washington.edu/phylip.html): we used PROTDIST and the JTT substitution model to generate the distance matrix based on the alignment, NEIGHBOR to infer the tree from the distance matrix, and SEQBOOT (1000 replicates) and CONSENSE to calculate the associated bootstrap values.

The ECH trees (Fig. S5) were calculated using Bayesian phylogenetic inference via MrBayes[46], given alignments calculated using MUSCLE. Both trees were calculated from four runs after 300,000 generations, with trees from the first 50,000 generations excluded from the estimation of the final tree.

All trees were visualized in Dendroscope[47].

### V. Extraction of distance matrices from networks, trees, and multiple sequence alignments

The central quantitative analysis in this work is the direct comparison of pairwise distance matrices between N-1 dimensional BLAST networks, two-dimensional displayed distances calculated by the Cytoscape 2.6[1] Organic layout, phylogenetic trees, and multiple sequence alignments. Here, BLAST E-values are the ideal distances that are indirectly captured by the Organic layout (this algorithm takes only node connectivity into account, not edge weights), while pairwise distances from a multiple sequence alignment are the ideal distances that are captured by phylogenetic trees. In order to compare a network, which contains cycles and many edges, to a tree, which has no cycles and few edges, we treat both networks and trees as graphs and calculate the shortest paths between each pair of sequences through the graph, using the −log_10_ edge E-values (BLAST), displayed edge lengths (Organic layout), and edge lengths from the Neighbor-Joining algorithm (trees) as edge weights. The shortest-paths matrix is calculated via the Floyd-Warshall algorithm, with the undirected networks represented as sets of pairs of opposite directed edges. Additionally, in a thresholded sequence similarity network, the distances between disconnected nodes are undefined; thus, analysis is only performed on the largest connected group of nodes for a given E-value threshold. In each of the figures associated with these calculations, the great majority of sequences are in the largest connected group of nodes. The multiple sequence alignment distances are calculated using the PHYLIP PROTDIST utility as described above.

### VI. Comparison of distance matrices and evaluation of statistical significance

The approach for comparing the above distance matrices and calculating the significance of their correlations is taken directly from Goh et al. 2000[48], which includes a detailed protocol. The reported statistics for each pair of matrices are R, Pearson's correlation coefficient; the estimated error, or bootstrap estimate of the standard deviation of the observed correlation; and the Z-score and corresponding P-value estimating the probability that a particular correlation between two matrices was obtained by chance.

### VII. Estimate of the effect of missing data

To evaluate how much sequence similarity networks change when some sequences are left out of the network, we removed 20% of the sequences at random from the Class A GPCR sequence set, and calculated Pearson's correlation between corresponding displayed distances based on the full 605-sequence set versus the 80% (484 sequences) set, as well as the underlying BLAST E-values. (The same Class A GPCR sequences are featured in Results Section III.) We used an E-value threshold of 1×10^−11^ to define the network. Derived statistics are based on ten replicates.

### VIII. Estimate of catalytic lysine in kinases

Each of the 513 human kinase domain sequences was aligned to either the PFAM Pkinase or Pkinase_Tyr family HMM[36]. If the best alignment had an E-value better than 1×10^−50^, indicating that the alignment was likely to be high quality, the amino acid aligning to the catalytic Lys in the model was identified. (The catalytic lysine is part of the “VAIK” motif in subdomain II of the kinase domain.) Whether this amino acid is the expected Lys or a different residue is mapped to the kinase superfamily network discussed in Results Section V.

### IX. Mapping taxonomic information to a sequence similarity network

NCBI maintains a hierarchical taxonomy database[49]; the database tables can be accessed at ftp.ncbi.nih.gov/pub/taxonomy/taxdump.tar.gz. These tables, which associate species names within a hierarchical taxonomic structure, were used to label network nodes with their species' taxonomic classification at various levels of a Tree of Life. This is illustrated at the end of Results Section V, in which each enoyl-CoA hydratase family sequence is colored according to its kingdom classification, or the superkingdom classification if there is no kingdom label. (For example, many parasites like *P. falciparum* are eukaryotes that have no kingdom classification.)

### X. External supplementary data

All data files generated in the analysis, including sequence files, HMMs, and network files, are available online at http://www.cgl.ucsf.edu/Research/cytoscape/SeqSimNet/. This website also includes a movie demonstrating how network topology changes with threshold, as well as IDs and accessions for all sequences specifically labeled in figures.

## Supporting Information

Table S1 Summary of network statistics: Correlation between organic laid-out network distances and the mathematically ideal BLAST E-value distances
(0.04 MB DOC)

Table S2 Comparison of mathematically ideal and displayed pairwise network distances between 51 human STE and WNK kinases
(0.05 MB DOC)

Table S3 Comparison of mathematically ideal and displayed pairwise distances between networks of the crotonase superfamily, using either full-length sequences or just the crotonase domain
(0.03 MB DOC)

Figure S1 Network distances are similar between the Organic and Cytoscape force-directed layout weighted by E-value
(2.13 MB PDF)

Figure S2 Comparison of network layout and clustering with BLASTCLUST
(3.85 MB PDF)

Figure S3 Graphic showing how network topology is affected by missing data. The correlation is high between the topology of the Class A GPCR network and networks with 20% of the sequences removed at random.
(0.75 MB PDF)

Figure S4 Comparison of trees and networks: STE and WNK kinases
(0.38 MB PDF)

Figure S5 The archaeal bECH ECH domain is more similar to the sECH domain than the non-archaeal bECH ECH domain
(1.11 MB PDF)

Figure S6 Asymmetery in BLAST E-values: How large is the difference between the E-values calculated between sequence pair A,B when A is used as query, or B is used as query?
(0.43 MB PDF)

Figure S7 Example percent identity and length of alignment quartile plots
(0.22 MB PDF)
