# Supplementary material for: Using Sequence Similarity Networks for Visualization of Relationships Across Diverse Protein Superfamilies
Source: PLoS One. 2009 Feb 3;4(2):e4345. doi: 10.1371/journal.pone.0004345 (PMC2631154; doi:10.1371/journal.pone.0004345)
Supplement: Table S1 — Summary of network statistics: Correlation between organic laid-out network distances and the mathematically ideal BLAST E-value distances (0.04 MB DOC) [file pone.0004345.s001.doc]

## Table S1. Summary of network statistics: Correlation between organic laid-out network distances and the mathematically ideal BLAST E-value distances

|  | Sequences^a^ | Edges^b^ | E-value Threshold^c^ | Correlation |
| --- | --- | --- | --- | --- |
| Amine-binding GPCRs (Fig. 2B) | 42 / 42 | 324 /  324 | 1×10^-33^  30% ID / 280 amino acids | R: 0.906 ± 0.034  Z: 11.87  P: 8.04 × 10^-33^ |
| STE and WNK kinases (Fig. S3B) | 51 / 51 | 821 /  821 | 1×10^-27^  30% ID / 270 amino acids | R: 0.846 ± 0.026  Z: 9.76  P: 8.18 × 10^-26^ |
| Enoyl-CoA hydratase family  (Fig. 6) | 329 / 410 | 15,723 / 16,054 | 1×10^-50^  40% ID / 260 amino acids | R: 0.873 ± 0.004  Z: 49.0  P: 0.0 |
| Kinase superfamily (Fig. 3) | 429 / 513 | 17,213 /  17,355 | 1×10^-25^  29% ID / 260 amino acids | R: 0.936 ± 0.003  Z: 40.9  P: 0.0 |
| Crotonase superfamily: domain-only sequences (Fig. 5C) | 825^d^ / 1170 | <40,014^d^ /  40,946 | 1×10^-29^  38% ID / 180 amino acids | R: 0.838 ± 0.002  Z: 31.5  P: 9.81 × 10^-219^ |
| Crotonase superfamily: full-length sequences (Fig. 5A) | 825^d^ / 1170 | <64,168^d^ /  74,470 | 1×10^-30^  33% ID / 250 amino acids | R: 0.867 ± 0.002  Z: 35.4  P: 9.68 × 10^-275^ |
| Class A Rhodopsin-like GPCRs (Fig. 4A) | 603 / 605 | 75,820 / 75,820 | 1×10^-11^  24% ID / 210 amino acids | R: 0.921 ± 0.002  Z: 54.4  P: 0.0 |
| GPCR suprafamily (Fig. 4B) | 766 / 766 | 140,544 / 140,544 | 1×10^-02^  22% ID / 120 amino acids | R: 0.924 ± 0.002  Z: 31.9  P: 1.99 × 10^-223^ |

^a^In the Sequences column, the first number reflects the number of sequences in the largest connected cluster that was considered for the correlation analysis. The second number reflects the total number of sequences in the dataset.

^b^The first number in the Edges column is the number of edges in the largest connected cluster that was considered in the calculations here. The second number reflects the total number of edges in the dataset.

^c^Listed with the network E-value threshold is the median percent identity and median alignment length for edges corresponding to the threshold E-value. These are the “worst” edges included in the analysis.

^d^The statistics on the crotonase superfamily networks are based on the distances between the 825 sequences in common in the large connected cluster between the full-length and domain-only networks. There are 974 nodes connected by 40,014 edges in the large connected cluster in the domain-only network, and 931 nodes connected by 64,168 edges in the full-length network.
