# Supplementary material for: Using Sequence Similarity Networks for Visualization of Relationships Across Diverse Protein Superfamilies
Source: PLoS One. 2009 Feb 3;4(2):e4345. doi: 10.1371/journal.pone.0004345 (PMC2631154; doi:10.1371/journal.pone.0004345)
Supplement: Table S2 — Comparison of mathematically ideal and displayed pairwise network distances between 51 human STE and WNK kinases (0.05 MB DOC) [file pone.0004345.s002.doc]

## Table S2. Comparison of mathematically ideal and displayed pairwise network distances between 51 human STE and WNK kinases

| A. BLAST E-values: (from pairwise alignments) | A.  BLAST E-values |  |  |
| --- | --- | --- | --- |
| B. Organic layout | R: 0.846 ± 0.026  Z: 9.76  P: 8.18 × 10^-23^ | B.  Organic layout |  |
| C. Neighbor Joining tree | R: 0.854 ± 0.026  Z: 10.63  P: 1.08 × 10^-26^ | R: 0.714 ± 0.026  Z: 8.23  P: 5.52 × 10^-19^ | C.  NJ tree |
| D. Distances from multiple sequence alignment | R: 0.851 ± 0.026  Z: 10.77  P: 2.50 × 10^-27^ | R: 0.713 ± 0.026  Z: 8.96  P: 1.59 × 10^-19^ | R: 0.974 ± 0.026  Z: 12.98  P: 7.71 × 10^-39^ |

Pearson’s correlations (R) and associated Z-scores (Z) and P-values (P) describing the similarity between the relative pairwise distances between 51 STE and WNK kinase domain sequences as assessed by (A) all shortest paths between –log_10_(BLAST E-values), (B) the shortest paths between sequences as displayed by a two-dimensional graph layout algorithm, (C) the shortest paths between sequences in a Neighbor-Joining tree, and (D) the relative pairwise distances calculated from a multiple sequence alignment. The pairwise BLAST E-values and the graph layout algorithm correspond to a network thresholded at an E-value of 1×10^-27^.
