# Supplementary material for: Using Sequence Similarity Networks for Visualization of Relationships Across Diverse Protein Superfamilies
Source: PLoS One. 2009 Feb 3;4(2):e4345. doi: 10.1371/journal.pone.0004345 (PMC2631154; doi:10.1371/journal.pone.0004345)
Supplement: Table S3 — Comparison of mathematically ideal and displayed pairwise distances between networks of the crotonase superfamily, using either full-length sequences or just the crotonase domain (0.03 MB DOC) [file pone.0004345.s003.doc]

## Table S3. Comparison of mathematically ideal and displayed pairwise distances between networks of the crotonase superfamily, using either full-length sequences or just the crotonase domain

| A. BLAST E-values: full-length sequences | A.  BLAST E-values: full-length |  |  |
| --- | --- | --- | --- |
| B. Organic layout: full-length sequences | R: 0.867 ± 0.002  Z: 35.4  P: 9.68 × 10^-275^ | B.  Organic layout:  full-length |  |
| C. Organic layout: domain only | R: 0.838 ± 0.002  Z: 31.5  P: 9.81 × 10^-219^ | R: 0.868 ± 0.002  Z: 33.2  P: 1.12 × 10^-241^ | C.  Organic layout:  domain only |
| D. BLAST E-values: domain only | R: 0.872 ± 0.002  Z: 33.0  P: 1.16 × 10^-238^ | R: 0.826 ± 0.002  Z: 30.7  P: 1.04 × 10^-207^ | R: 0.918 ± 0.002  Z: 33.4  P: 4.42 × 10^-245^ |

Pearson’s correlations (R) and associated Z-scores (Z) and P-values (P) describing the similarity between the relative pairwise distances between 825 crotonase superfamily sequences found in common in the large connected clusters in Fig. 5A and 5C, as assessed by all shortest paths between –log_10_(BLAST E-values) using the full-length or domain-only sequence (A and D), and the shortest paths between full-length and domain-only sequences as displayed by a two-dimensional graph layout algorithm (B and C). These networks were thresholded at a BLAST E-value of 1×10^-30^ (full length) or 1×10^-29^ (domain only). 1×10^-30^ corresponds to a median of 33% identity over alignments of 250 amino acids, and 1×10^-29^ corresponds to a median of 38% identity over alignments of 180 amino acids. Note that the layouts (B and C) are visual representations of the underlying distances in A and D, respectively. A and D cannot be visualized exactly in fewer than N-1 dimensions.
