# Supplementary material for: Using Sequence Similarity Networks for Visualization of Relationships Across Diverse Protein Superfamilies
Source: PLoS One. 2009 Feb 3;4(2):e4345. doi: 10.1371/journal.pone.0004345 (PMC2631154; doi:10.1371/journal.pone.0004345)

Fig S1. Network distances are similar between the Organic and Cytoscape force-directed layout weighted by E-value.

This page: Statistics (A) describing the comparison of the kinase network from Fig. 3 laid out using the Organic layout (B) or the Cytoscape force-directed layout weighted by edge E-value (C).
Following page: Statistics (D) describing the comparison of the amine GPCR network from Fig. 2B laid out using the Organic layout (E) or the Cytoscape force-directed layout weighted by edge E-value (F).

A.

A. BLAST E-values	A.<br>BLAST E-values	
B. Organic layout	R: 0.934 ± 0.003<br>Z: 41.2<br>P: 0.0	B.<br>Organic layout
C. Cytoscape force-directed layout weighted by E-value	R: 0.895 ± 0.003<br>Z: 35.3<br>P: 7.02×10^{-273}	R: 0.900 ± 0.003<br>Z: 36.8<br>P: 2.92×10^{-297}

Pearson's correlations (R) and associated Z-scores (Z) and P-values (P) describing the similarity between the relative pairwise distances between 419 human kinase domain sequences in common as assessed by (A) all shortest paths between $-\log_{10}$(BLAST E-values), (B) the shortest paths between sequences as displayed by the Organic two-dimensional graph layout algorithm, and (C) the shortest paths between sequences in the two dimensional Cytoscape force-directed layout, weighted by BLAST E-value. The pairwise BLAST E-values and the graph layout algorithm correspond to a network thresholded at an E-value of 1×10^{-25}.

B. Organic Layout

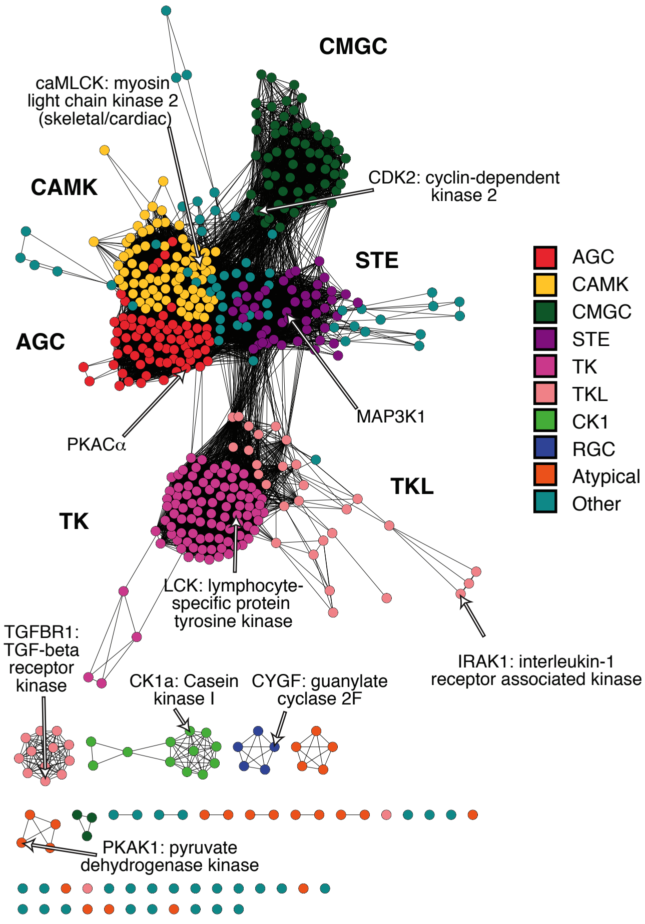

C. Force-directed layout weighted by E-value

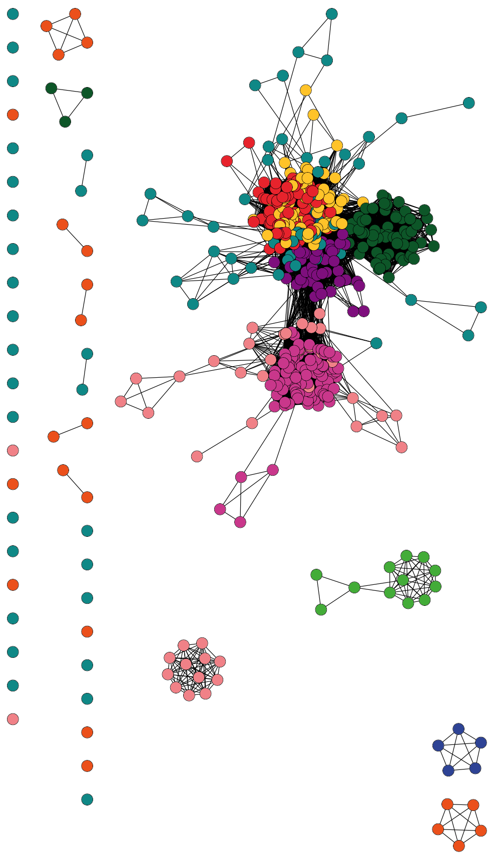

Fig S1. Continued

D.

	A. BLAST E-values	B. Organic layout
A. BLAST E-values		
B. Organic layout	R: 0.906 ± 0.034<br>Z: 11.87<br>P: 8.04×10^{-33}	
C. Cytoscape force-directed layout weighted by E-value	R: 0.850 ± 0.034<br>Z: 9.52<br>P: 8.78×10^{-22}	R: 0.877 ± 0.035<br>Z: 10.16<br>P: 1.47×10^{-24}

Pearson's correlations (R) and associated Z-scores (Z) and P-values (P) describing the similarity between the relative pairwise distances between 42 amine GPCR domain sequences in common as assessed by (A) all shortest paths between $-\log_{10}$(BLAST E-values), (B) the shortest paths between sequences as displayed by the Organic two-dimensional graph layout algorithm, and (C) the shortest paths between sequences in the two dimensional Cytoscape force-directed layout, weighted by BLAST E-value. The pairwise BLAST E-values and the graph layout algorithm correspond to a network thresholded at an E-value of 1×10^{-33}.

E. Organic Layout

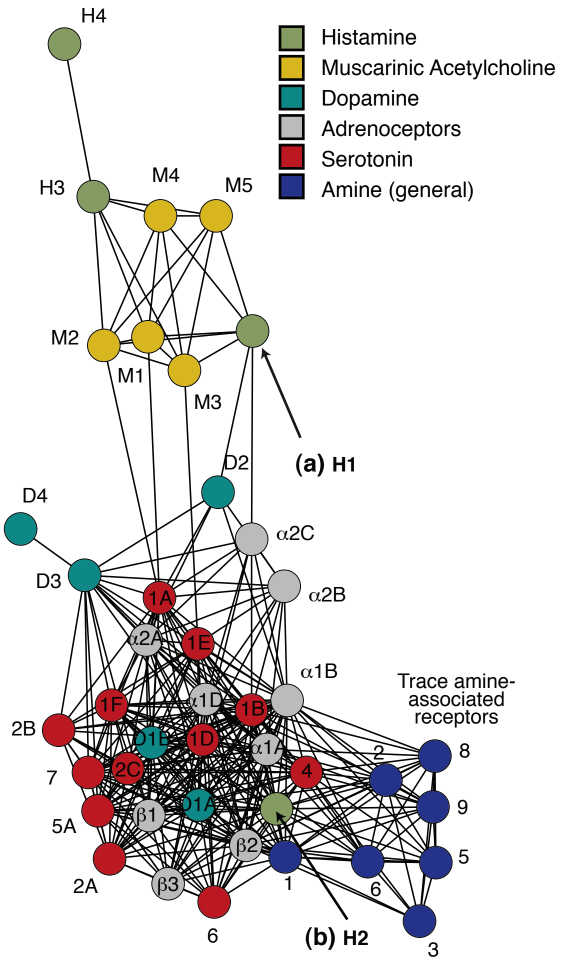

F. Force-directed layout weighted by E-value

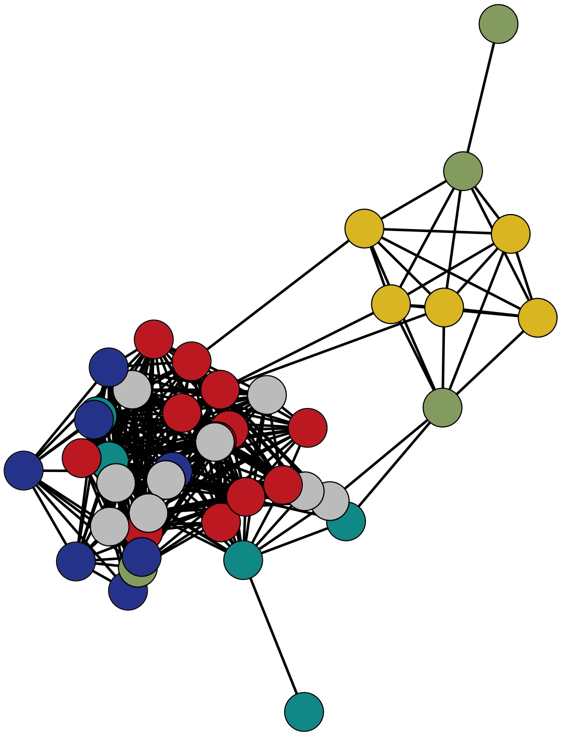

Supplement: Figure S1 — Network distances are similar between the Organic and Cytoscape force-directed layout weighted by E-value (2.13 MB PDF) [file pone.0004345.s004.pdf]
