# Supplementary material for: Using Sequence Similarity Networks for Visualization of Relationships Across Diverse Protein Superfamilies
Source: PLoS One. 2009 Feb 3;4(2):e4345. doi: 10.1371/journal.pone.0004345 (PMC2631154; doi:10.1371/journal.pone.0004345)
Supplement: Figure S2 — Comparison of network layout and clustering with BLASTCLUST (3.85 MB PDF) [file pone.0004345.s005.pdf]

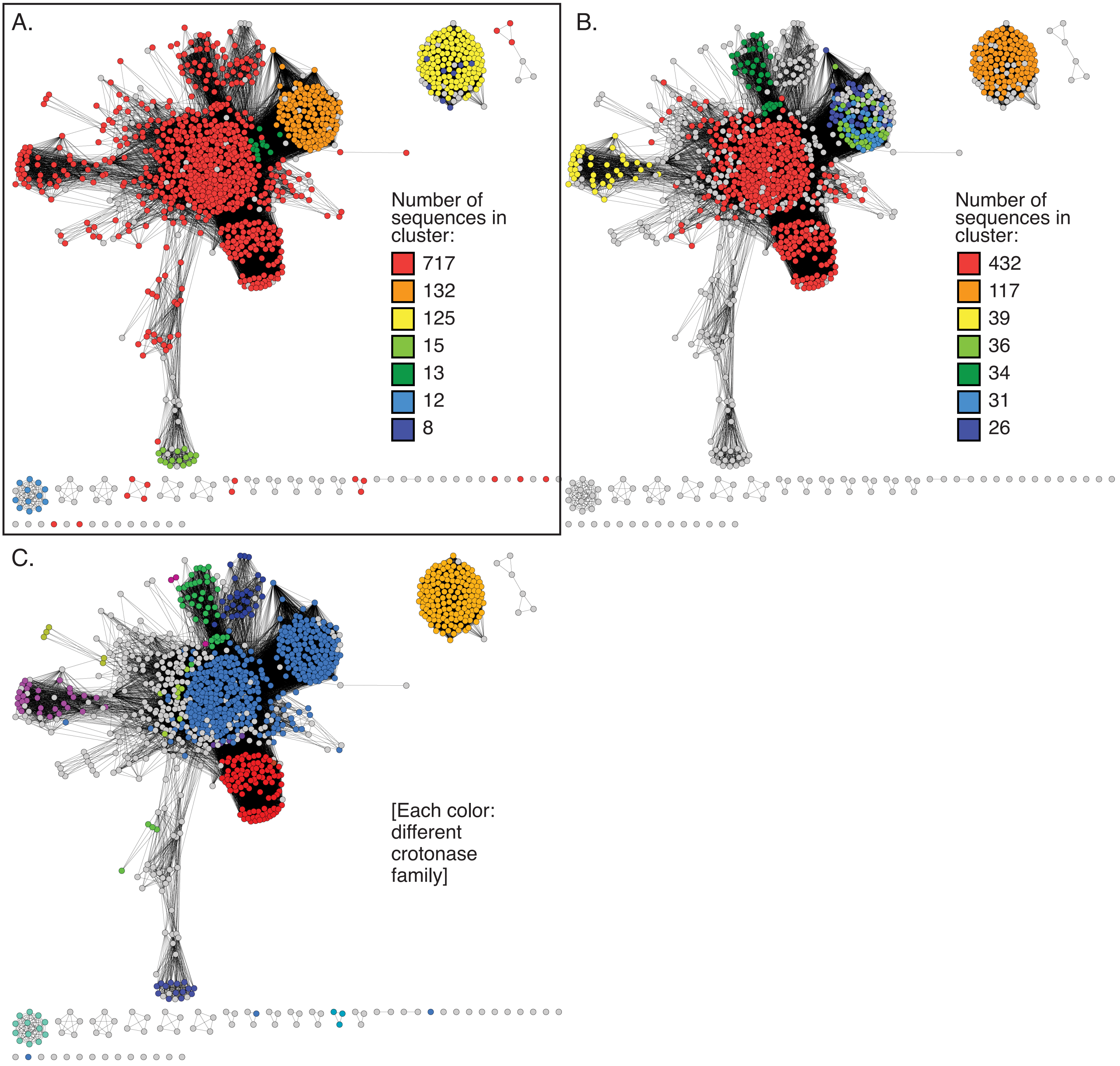

Fig. S2. Comparison of network layout and clustering with BLASTCLUST
The displayed networks all describe the pairwise relationships between 1,170 sequences from the crotonase superfamily, thresholded at BLAST E-value of $1x10^{-30}$. The worst edges displayed correspond to a median of 33% identity over alignments of 250 residues. Note that there is no alignment coverage requirement, and half of the edges with an E-value equal to 1x10-30 have a percent identity >33%. **A.** Here, the crotonase superfamily network is shown colored by membership in the seven most populated clusters as determined by the NCBI BLASTCLUST program, using a cut-off of 33% identity and requiring 90% coverage. **B.** Sequences are colored according to BLASTCLUST clusters using a cut-off of 40% identity and requiring 90% coverage. **C.** As a reference, the same network is shown colored according to family membership, based on catalytic function, as in Fig. 5A.
