# Supplementary material for: Using Sequence Similarity Networks for Visualization of Relationships Across Diverse Protein Superfamilies
Source: PLoS One. 2009 Feb 3;4(2):e4345. doi: 10.1371/journal.pone.0004345 (PMC2631154; doi:10.1371/journal.pone.0004345)
Supplement: Figure S3 — Graphic showing how network topology is affected by missing data. The correlation is high between the topology of the Class A GPCR network and networks with 20% of the sequences removed at random. (0.75 MB PDF) [file pone.0004345.s006.pdf]

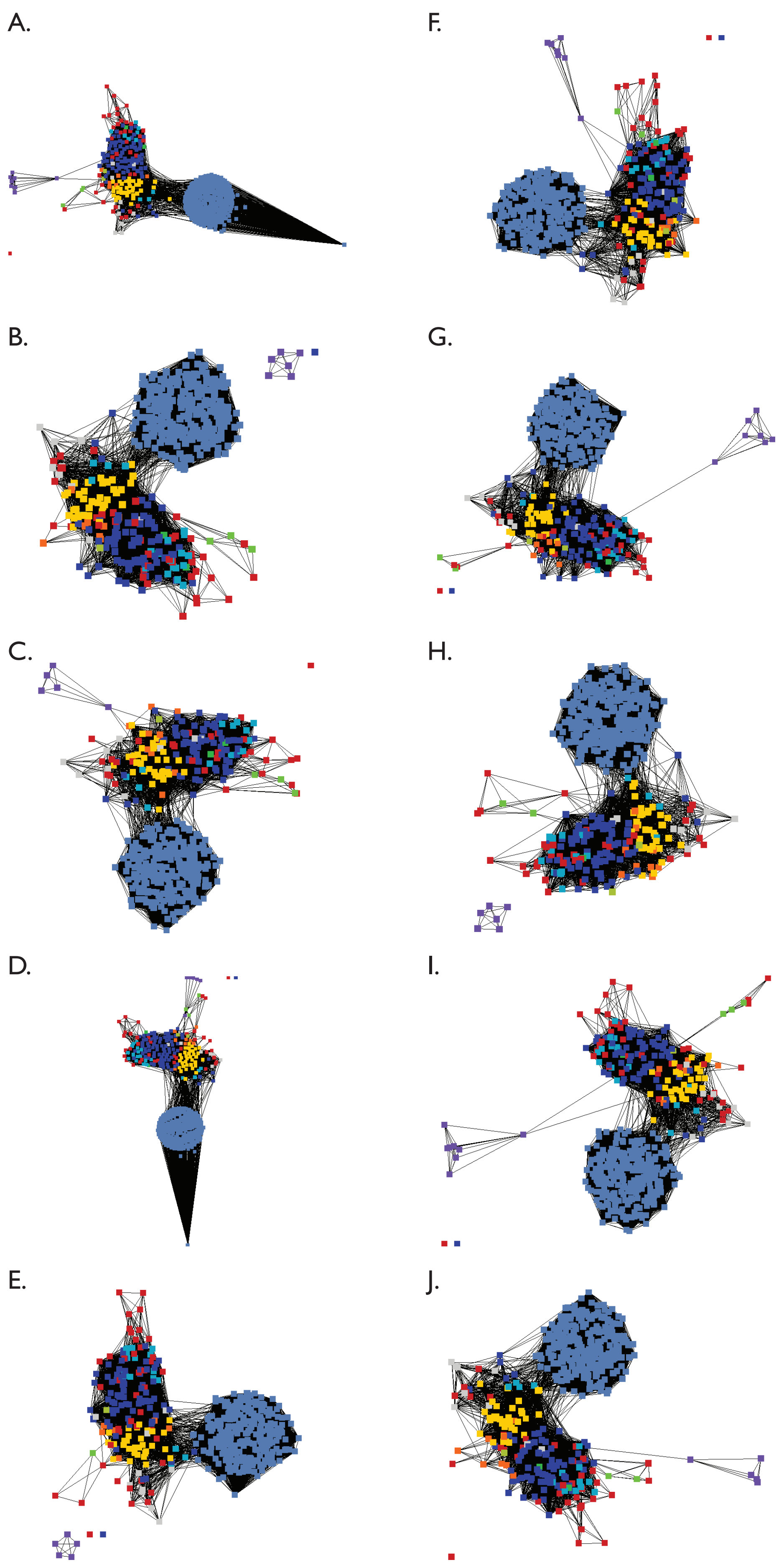

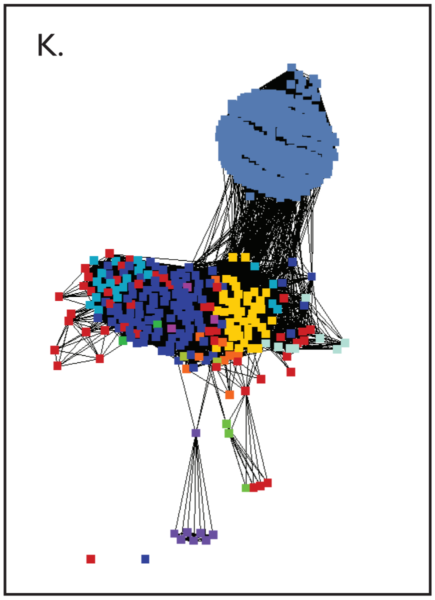

Supplementary Fig. 3. The correlation is high between the topology of the Class A GPCR network and networks with 20% of the sequences removed at random.
To evaluate how much sequence similarity networks change when some sequences are left out of the network, we removed 20% of the sequences at random from the Class A GPCR sequence set (Fig. 4A), and calculated Pearson's correlation between corresponding displayed distances based on the full 605-sequence set versus the 80% (484 sequences). We used the same E-value cutoff (1e-11) used in Fig. 4A). Derived statistics discussed in the results section are based on the ten replicates, shown in A-J. The full 605-sequence network is shown in K. (The correlation between displayed distances in K and A-J was 0.892 on average.) Visually, the relative positioning of different groups is consistent.
