# Supplementary material for: Using Sequence Similarity Networks for Visualization of Relationships Across Diverse Protein Superfamilies
Source: PLoS One. 2009 Feb 3;4(2):e4345. doi: 10.1371/journal.pone.0004345 (PMC2631154; doi:10.1371/journal.pone.0004345)
Supplement: Figure S4 — Comparison of trees and networks: STE and WNK kinases (0.38 MB PDF) [file pone.0004345.s007.pdf]

Fig. S4. Comparison of trees and networks: STE and WNK kinases

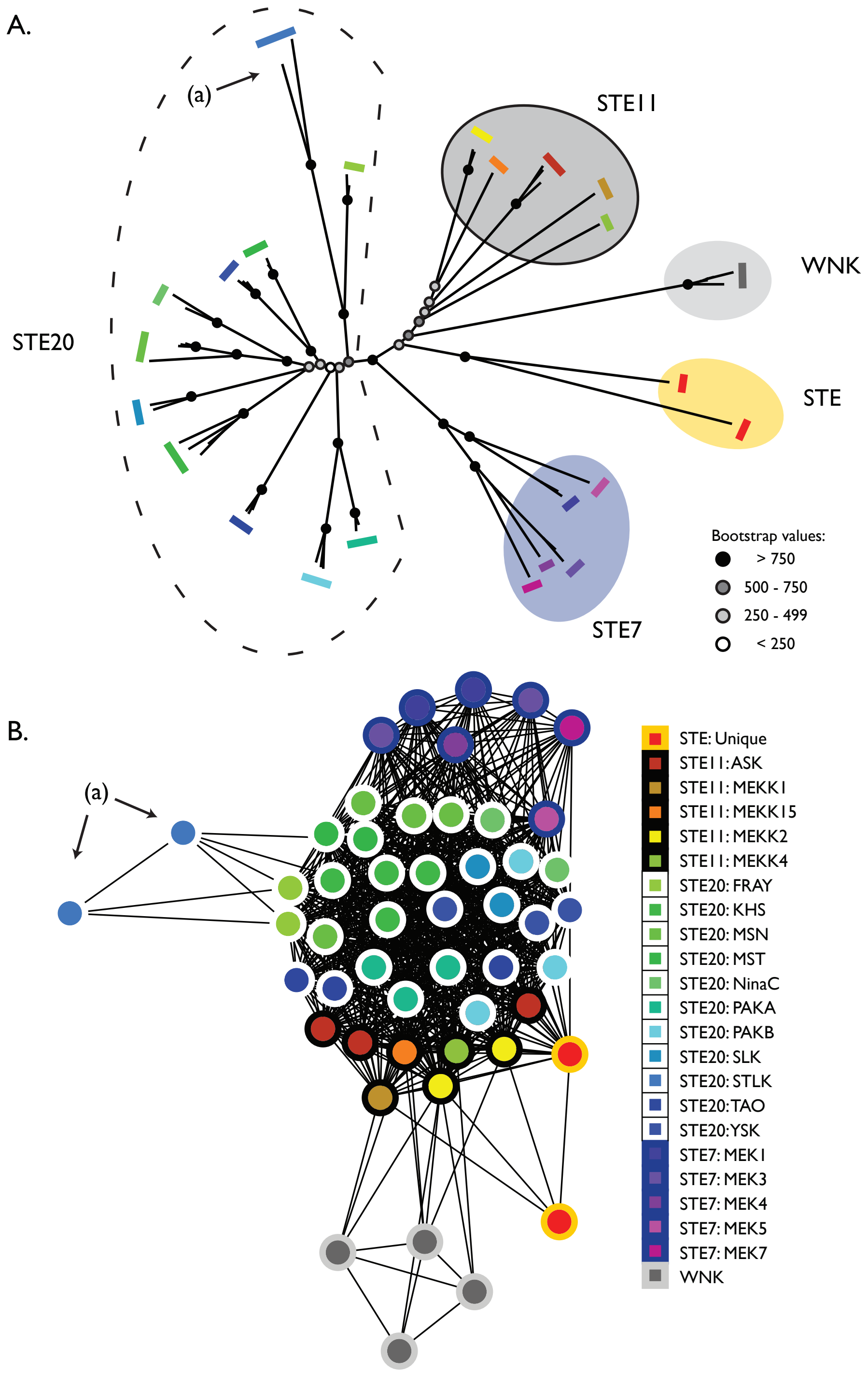

Fig. S4. Comparison of trees and networks: STE and WNK kinases. **A.** Neighbor-Joining tree describing the interrelationships of 51 kinase domains. **B.** Sequence similarity network including the same 51 sequences as in A. This network was thresholded at an E-value of $1x10^{-27}$ and contains 821 edges; the worst edges displayed correspond to a median of 30% identity over an alignment length of 270 amino acids. The node borders are colored according to superclass (e.g., STE20, STE7) and the node fill corresponds to their subclass. The sequences labeled (a) are discussed in the text. See Table S2 for a quantitative comparison of the two representations.
