# Supplementary material for: Using Sequence Similarity Networks for Visualization of Relationships Across Diverse Protein Superfamilies
Source: PLoS One. 2009 Feb 3;4(2):e4345. doi: 10.1371/journal.pone.0004345 (PMC2631154; doi:10.1371/journal.pone.0004345)
Supplement: Figure S5 — The archaeal bECH ECH domain is more similar to the sECH domain than the non-archaeal bECH ECH domain (1.11 MB PDF) [file pone.0004345.s008.pdf]

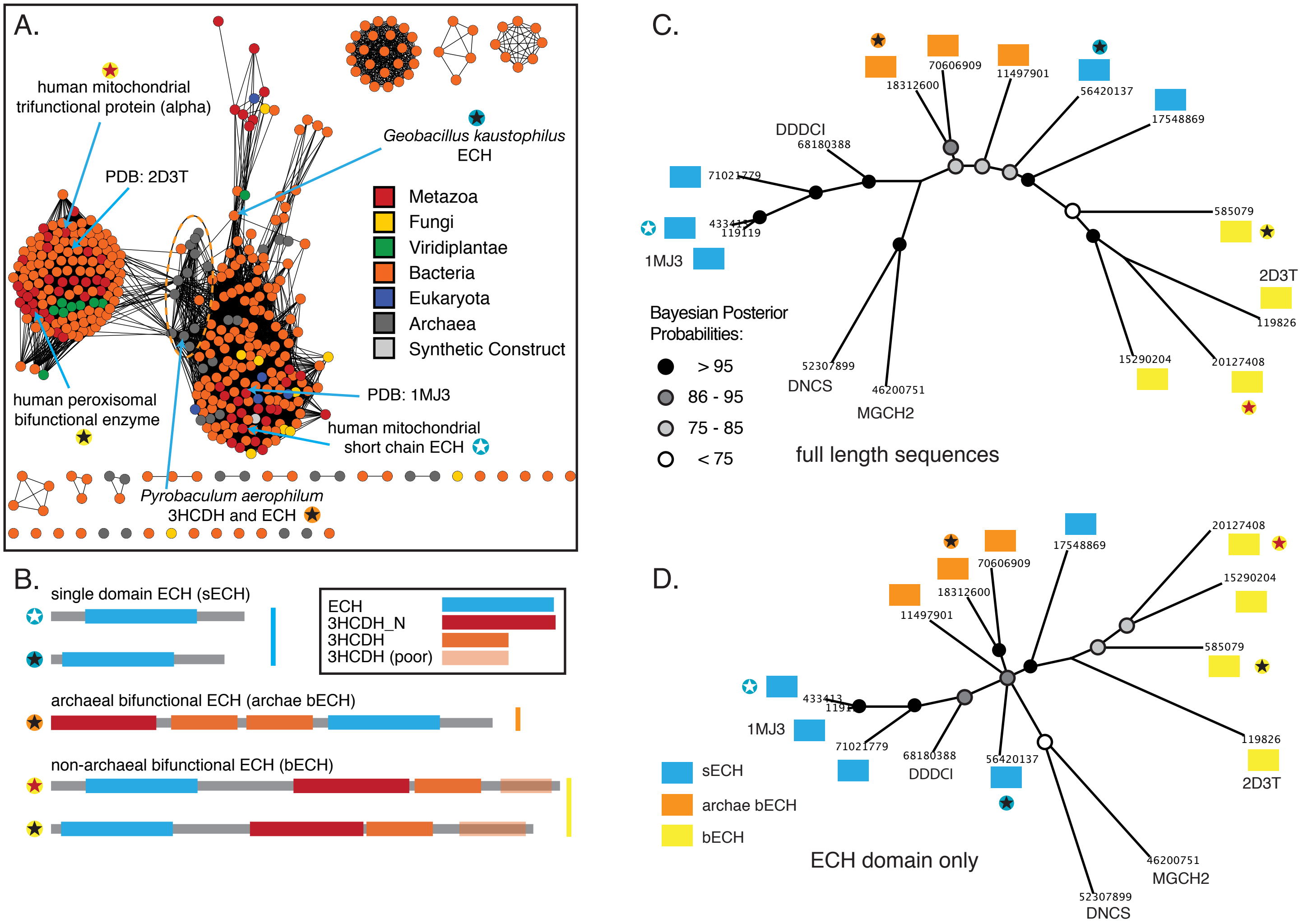

Fig. S5. The archaeal bECH ECH domain is more similar to the sECH domain than the non-archaeal bECH ECH domain

Here, Fig. 5B-C are repeated as A and B and displayed next to phylogenetic trees containing representative sequences. Each of the three categories of ECH enzyme (sECH, archaeal bECH, and non-archaeal bECH) shares significant sequence similarity; in particular, the *P. aerophilum* bECH ECH domain is 41% identical to the human mitochondrial short chain ECH (sECH), and 37% identical to the human peroxisomal bifunctional enzyme (bECH).

A. The displayed network contains all 410 enoyl-CoA hydratases from the crotonase superfamily network in Fig. 5A. The network is thresholded at a BLAST E-value of $1x10^{-50}$; the worst edges displayed correspond to a median of 40% identity over alignments of 260 amino acids. Network nodes are colored by species kingdom (Fungi, Metazoa, Viridiplantae) or superkingdom (Bacteria, Eukaryota, Archaea). The archaebacterial bifunctional enzymes are marked with a dashed oval in A. **B.** Representative domain structures for the three major classes of enoyl-CoA hydratase-containing sequences, with domains defined using PFAM HMMs. **C.** Bayesian phylogenetic tree of representative full-length sequences from the network in A and three other crotonase superfamily enzymes. **D.** Bayesian phylogenetic tree based on just the ECH domains from the sequences in C. The displayed IDs are NCBI protein sequence general identifiers.
