# Supplementary material for: Using Sequence Similarity Networks for Visualization of Relationships Across Diverse Protein Superfamilies
Source: PLoS One. 2009 Feb 3;4(2):e4345. doi: 10.1371/journal.pone.0004345 (PMC2631154; doi:10.1371/journal.pone.0004345)
Supplement: Figure S6 — Asymmetery in BLAST E-values: How large is the difference between the E-values calculated between sequence pair A,B when A is used as query, or B is used as query? (0.43 MB PDF) [file pone.0004345.s009.pdf]

Fig. S6. Asymmetry in BLAST E-values: How large is the difference between the E-values calculated between sequence pair A,B when A is used as query, or B is used as query?

A. Larger E-value differences tend to occur at more significant E-value scores

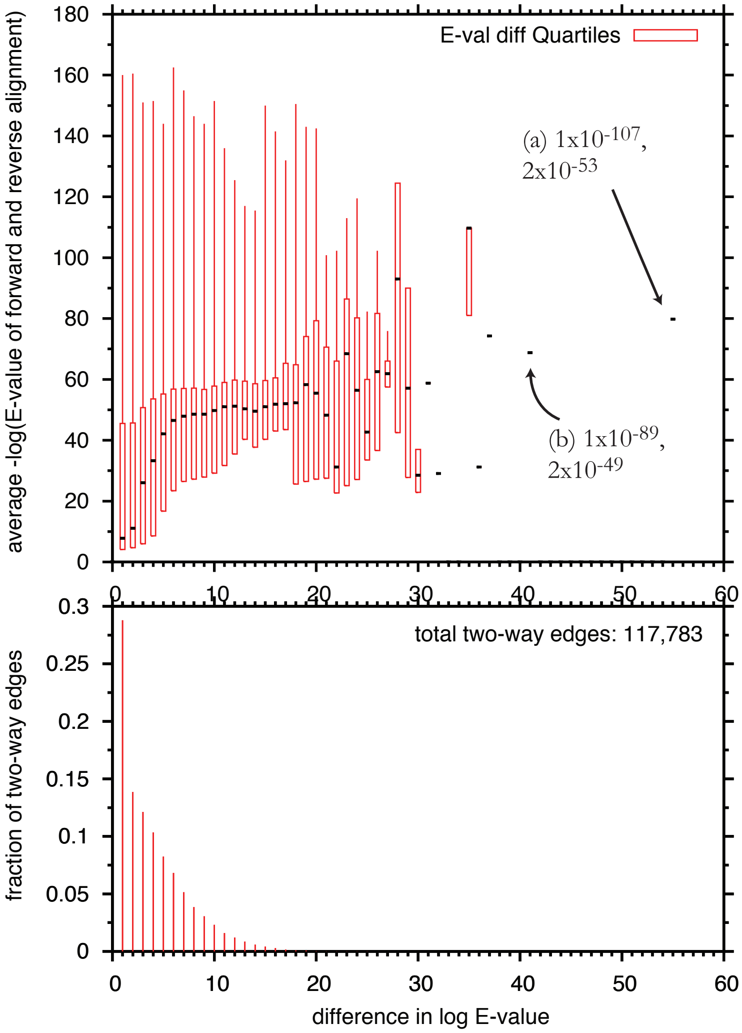

S6A is a quartile plot summary of the "forward" and "reverse" E-values assigned to 117,783 edges from the 766-sequence GPCR suprafamily network depicted in Fig. 4B. This network was thresholded at an E-value of $1x10^{-2}$. The plot shows that most of the pairwise relationships had a small change in the E-value when the query sequence was swapped; in the smallest difference and most populated bin, where the log E-value difference is between 0 and 1 (ie, forward: $1.7x10^{-8}$; reverse: $8.8x10^{-9}$) the range of E-values at which this magnitude of difference occurs is very broad, but concentrated at less significant E-values, most likely because there are always more lower-significance edges than high-significance edges in a thresholded sequence similarity network describing a real data set. However, the largest differences tend to occur at very significant E-values; note the increase in the median average E-value of the pair as the difference increases. Extremely large differences are very rare, but exist. Two of these are singled out in S6A: the difference corresponding to (a) resulted from one sequence being highly masked by the 'seg' low-complexity filter when it was used as query, but not the other. Whether masking is likely to help or hurt more will depend on the data set. In (b), when one sequence was used as query, the alignment was extended much further than when the other sequence served as query.

B. When an edge is defined by only one sequence query, it is usually a low-significance relationship

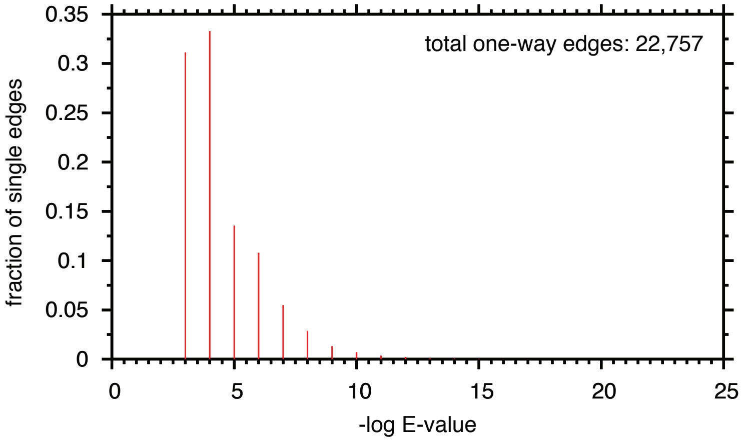

S6B is a plot of the 22,757 edges in the same GPCR suprafamily network that are based only on one query resulting in an E-value better than the $1x10^{-2}$ threshold. Although there are a few disconcerting edges with "good" E-values that did not pass the threshold when the other sequence was used as query, these "one-way" edges are far fewer than the 117,783 that represent "forward" and "reverse" E-values, and they are mostly at low levels of similarity. Here, there are single edges at $1x10^{-21}$ and $2x10^{-24}$ that are not visible in the plot.
