# Supplementary material for: Using Sequence Similarity Networks for Visualization of Relationships Across Diverse Protein Superfamilies
Source: PLoS One. 2009 Feb 3;4(2):e4345. doi: 10.1371/journal.pone.0004345 (PMC2631154; doi:10.1371/journal.pone.0004345)
Supplement: Figure S7 — Example percent identity and length of alignment quartile plots (0.22 MB PDF) [file pone.0004345.s010.pdf]

Fig. S7. Example percent identity and length of alignment quartile plots

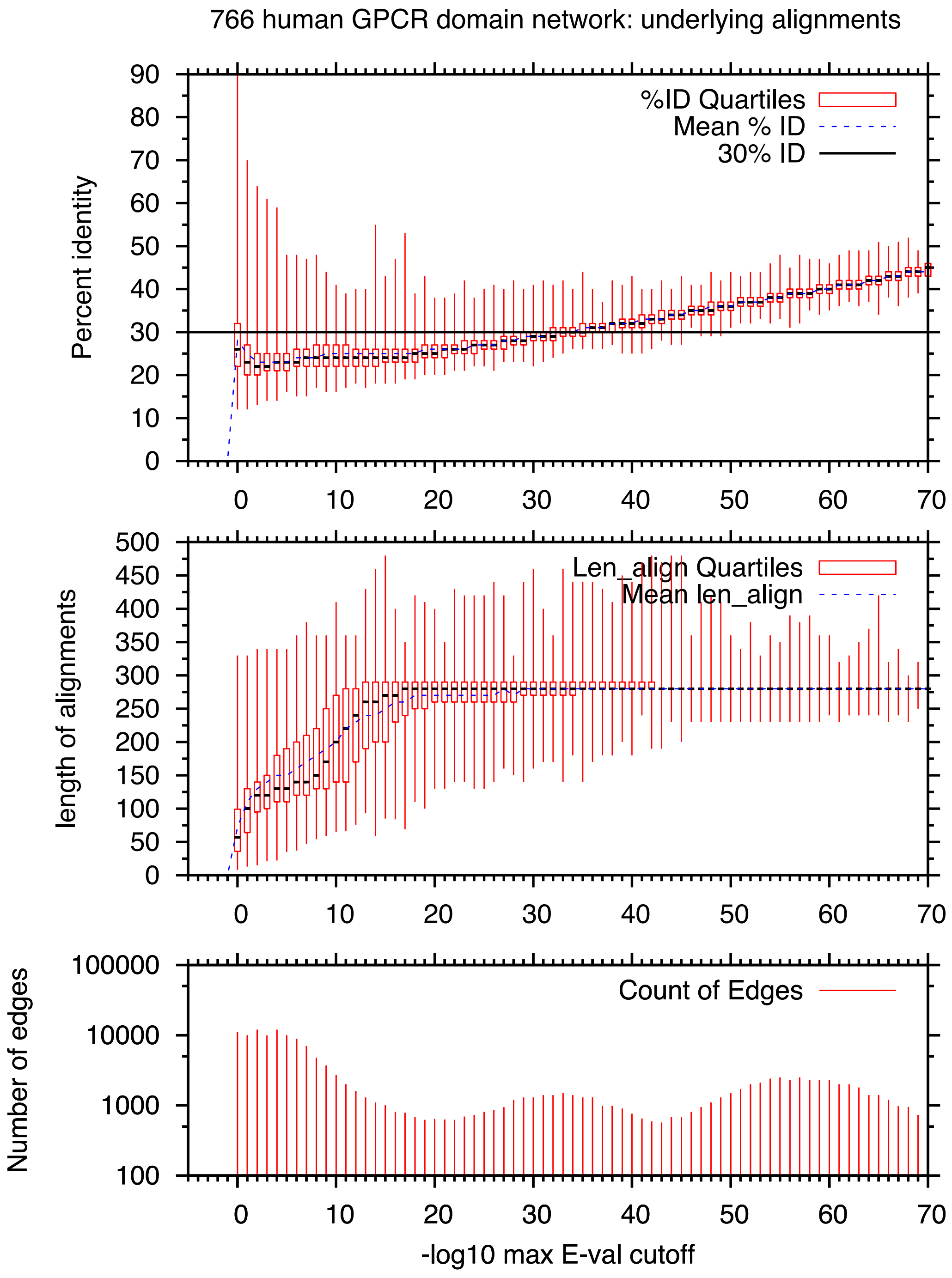


**Fig. S7.** This series of plots describes the alignments at different E-values associated with the 766-sequence GPCR suprafamily that is depicted using a $1x10^{-2}$ E-value threshold in Fig. 4B. Observing the range of percent identities and alignment lengths associated with each E-value range is a key step in deciding which thresholded networks to examine in more detail. For example, perhaps only networks based on alignments that cover at least the length of the domain in common and have greater than 30% sequence identity are of interest.
